# Privileged Scaffold Hybridization in the Design of Carbonic Anhydrase Inhibitors

**DOI:** 10.3390/molecules29184444

**Published:** 2024-09-19

**Authors:** Daniela Secci, Erica Sanna, Simona Distinto, Alessia Onali, Antonio Lupia, Laura Demuru, Giulia Atzeni, Rita Meleddu, Filippo Cottiglia, Andrea Angeli, Claudiu T. Supuran, Elias Maccioni

**Affiliations:** 1 Department of Life and Environmental Sciences, University of Cagliari, Cittadella Universitaria di Monserrato, Monserrato, 09042 Cagliari, Italy; daniela.secci@ffa.uni-lj.si (D.S.); erica.sanna@unica.it (E.S.); s.distinto@unica.it (S.D.); alessia.onali@unica.it (A.O.); antonio.lupia@unica.it (A.L.); laura.demuru2@unica.it (L.D.); giulia.atzeni@unica.it (G.A.); filippo.cottiglia@unica.it (F.C.); elias.maccioni@unica.it (E.M.); 2 Net4Science S.r.l, Università Degli Studi “Magna Græcia” di Catanzaro, 88100 Catanzaro, Italy; 3 Dipartimento Neurofarba, Sezione di Scienze Farmaceutiche, Università degli Studi di Firenze, Sesto Fiorentino, 50019 Florence, Italy; andrea.angeli@unifi.it

**Keywords:** carbonic anhydrases inhibitors, scaffold hybridization, benzenesulfonamide-based zinc binders

## Abstract

Human Carbonic Anhydrases (hCA) are enzymes that contribute to cancer’s development and progression. Isoforms IX and XII have been identified as potential anticancer targets, and, more specifically, hCA IX is overexpressed in hypoxic tumor cells, where it plays an important role in reprogramming the metabolism. With the aim to find new inhibitors towards IX and XII isoforms, the hybridization of the privileged scaffolds isatin, dihydrothiazole, and benzenesulfonamide was investigated in order to explore how it may affect the activity and selectivity of the hCA isoforms. In this respect, a series of isatin thiazolidinone hybrids have been designed and synthesized and their biological activity and selectivity on hCA I, hCA II, hCA IX, and hCA XII explored. The new compounds exhibited promising inhibitory activity results on isoforms IX and XII in the nanomolar range, which has highlighted the importance of substituents in the isatin ring and in position 3 and 5 of thiazolidinone. In particular, compound **5g** was the most active toward hCA IX, while **5f** was the most potent inhibitor of hCA XII within the series. When both potency and selectivity were considered, compound **5f** appeared as one of the most promising. Additionally, our investigations were supported by molecular docking experiments, which have highlighted the putative binding poses of the most promising compound.

## 1. Introduction

**Figure 1:**
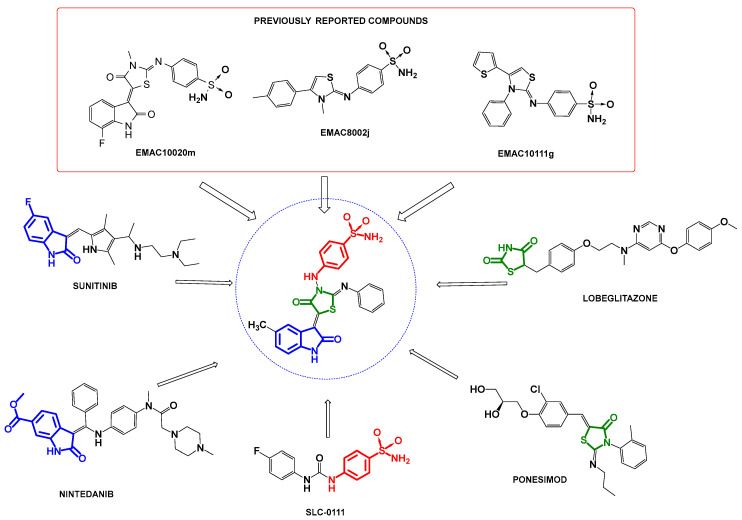
Representative previously reported compounds and design of **5a**–**i** derivatives obtained by combining different fragments of biologically active molecules.

Cancer is a complex disease, where multiple implicated genes may simultaneously display mutations that interfere with the physiological processes of cells and cause a rise in malignant phenotype [1,2]. Therefore, to focus pharmacological attention on the right targets, it is essential to identify the aberrant proteins and enzymes involved in the molecular pathways of cancer processes. Accordingly, medicinal chemists have concentrated their efforts on several specific and validated targets in order to identify new molecules and approaches for a more successful cancer treatment. In this respect, the contribution of human Carbonic Anhydrases (hCA), especially hCA IX and XII isozymes, in the development of cancer has been widely investigated and validated [3,4,5]. CA enzymes are ubiquitous metalloenzymes that contain a zinc ion in the active site [6]. They catalyze the reversible conversion of carbon dioxide into bicarbonate ions and protons [7,8], regulating pH and other relevant physiological processes. Mammals encode for α-CAs, further divided in 16 isoforms differing for the sequence, tissue localization, expression, and activity [9,10,11]. The majority of healthy tissues do not express isoform IX [12]. On the other hand, hypoxic and malignant tumors with more aggressive subtypes usually exhibit high expression levels of hCA IX. While some tumors express both hCA IX and hCA XII, hCA XII is more commonly linked to a well-differentiated, less aggressive phenotype [13,14,15]. Several experimental studies have revealed that hypoxia and pH regulation are critical for the survival and growth of tumor cells [16,17]. As a consequence, hCA IX and XII inhibitors and theranostics were thoroughly explored as possible anticancer drugs [18,19,20,21,22]. The two primary approaches for targeting tumor-associated hCAs for cancer therapy are the production of monoclonal antibodies and the development and synthesis of small molecules that selectively inhibit hCAs XI and XII [23]. In this respect benzenesulfonamide-based derivatives have been widely explored, due to the ability of the sulfonamide to coordinate the zinc ion in the catalytic cavity of hCAs [24,25,26,27]. However, due to the potent interaction established by the zinc binder sulfonamide moiety and the hCA isozymes, selectivity is often an issue, and further molecular decoration is often needed to seek selectivity [28]. Indeed, the design of isoform-selective benzenesulfonamide-based hCAs inhibitors requires the addition of further molecular features capable of selectively interacting with the most external aminoacidic residues of the catalytic cleft, where the higher diversity among the hCAs isoforms can be found. As a continuation of ongoing research (series **EMAC10020**, **EMAC8002**, and **EMAC10111**) [10,29,30,31], our efforts have focused on developing a series of small molecules via the hybridization of privileged scaffolds. Indeed, the conjugation of privileged scaffolds from anticancer agents may lead to the identification of novel molecular entities that might also combine the biological potential of the origine compounds and exert a multitarget activity profile [32,33]. With respect to previously investigated compounds, the nature and the substitution pattern on the central heterocyclic core has been modified, as depicted in Figure 1. Moreover, privileged scaffolds originating from diverse anticancer agents have been conjugated in one single molecule. The compounds have been fully characterized and tested on hCAs. Furthermore, ligand–protein interactions have been predicted using molecular docking experiments.

## 2. Results and Discussion

### 2.1. Chemistry

As a result of an ongoing project, a series of molecules with a thiazolidinone/isatin scaffold, namely compounds **5a**–**i,** have been synthesized. Hence, starting from the previously synthesized compound **EMAC10020m**, which showed significant inhibitory activity against hCA IX and XII in the nanomolar range and an advantageous profile of selectivity between hCA IX and other hCA isozymes [10], new structural modifications were introduced to investigate their effect on both activity and selectivity. The added modifications considered the structural differences between the target and off-target hCA isoforms, aiming at the goal of preserving potency while increasing selectivity against the IX and XII isoforms. With respect to compound **EMAC10020m**, the combination of the indolinone, thiazolidinone, and benzenesulfonamide scaffolds was preserved, although with substantial modifications that may lead to the further development of derivatives such as multitarget agents. More in detail (Figure 1), the indolinone scaffold is a common structural feature in clinically approved VEGFR inhibitors such as sunitinib and nintedanib. In particular, the nitrogen and the carbonyl oxygen in positions 1 and 2 of the indolinone moiety are known to establish a H-bond network with the hinge region of the VEGFR kinase site, in the proximity of the ATPase center [34,35].

As for sunitinib and nintedanib, position 3 of the indolinone ring of compounds **5a**–**i** is substituted with a methylidene moiety enclosed in the thiazolidinone heterocycle [36]. This latter heterocycle is also present in several approved drugs and, in particular, in lobeglitazone and in ponesimod (Figure 1), two FDA-approved drugs for the treatment of diabetes and relapsing multiple sclerosis. Despite the facts that both lobeglitazone and ponesimod are apparently not indicated for cancer treatment, we must consider that the former is a PPARγ agonist, while the latter is a sphingosine 1-phosphate receptor modulator (S1Pr)m, and both PPARγ and S1Pr have been reported to have a role in angiogenesis, proliferation, and in the apoptosis escape in cancer [37,38]. The benzenesulfonamide moiety was selected due to its key zinc-binding role and becouse it is a common feature of carbonic anhydrase inhibitors (CAIs), such as SLC-0111, an inhibitor of hCAIX under clinical investigation for the treatment of advanced solid tumors [39].

Moreover, to further explore the structure activity relationships of these hybrid molecules, we focused our efforts on positions 2 and 3 of the thiazolidinone ring. Indeed, with respect to the previously reported **EMAC10020m**, in the **5a**–**i** series, the benzene sulfonamide moiety was moved from position 2 to position 3 of the heterocyclic ring, and a phenyl ring was introduced in position 2. The latter modification increases the steric hindrance of the molecule tail, thus boosting interactions between the ligands and the aminoacidic residues at the entrance of the activity sites of isoforms IX and XII.

**Scheme 1:**
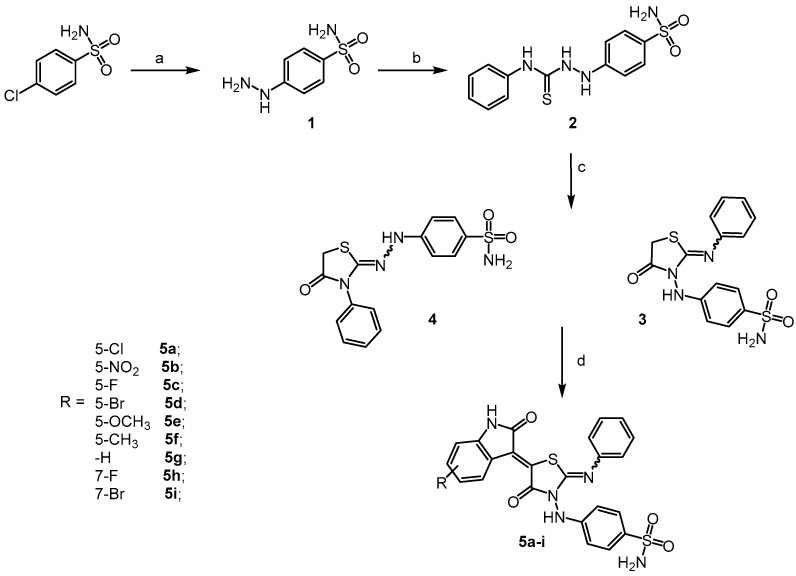
Reaction conditions: (a) hydrazine monohydrate, MW, 40 min; (b) phenyl isothiocyanate, ethanol, reflux, overnight; (c) ethyl bromoacetate, sodium acetate, ethanol, reflux, 4 h; (d) appropriate isatin, sodium acetate, acetic anhydride, and acetic acid, reflux for 16 h.

The **5a**–**i** series has been synthesized according to the multi-step synthetic approach shown in Scheme 1.

The first step of the synthetic pathway consists of the reaction of 4-chlorobenzenesulfonamide with hydrazine hydrate under microwave irradiation. This reaction was devised by modifying a literature-reported method [40]. By this new approach, the reaction time was dramatically reduced compared to that reported in the literature (from 20 h to 1.5 h), without affecting the reaction yield. The resulting 4-hydrazineylbenzenesulfonamide was further reacted with phenyl isothiocyanate to produce *N*-phenyl-2-(4-sulfamoylphenyl)hydrazine-1-carbothioamide **2**. By reacting **2** with ethyl bromoacetate, the formation of thiazolidinones **3** and **4** was observed. Indeed, two regioisomers were formed. Compound **3** was the major product, with a ratio 3/4 higher than 85/25. Nevertheless, the two regioisomers were characterized by means of NMR spectroscopy experiments.

In Table 1 and Table 2, the ^13^C NMR chemical shifts are reported for regioisomers **3** and **4,** respectively. When comparing the two regioisomers, in compound **3**, the chemical shift of C2a is moved to lower fields (153.13 ppm, Table 1), as if it binds the imine, while, in the case of compound **4,** the same carbon atom of the phenyl ring, namely C3a, is shifted at 133.73 ppm (Table 2).

To better clarify the structure of the two compounds, the ^1^H, ^13^C HMBC (Heteronuclear Multiple-Bond Correlation) spectra of both thiazolidinone regioisomers **3** and **4** were recorded.

**Figure 2:**
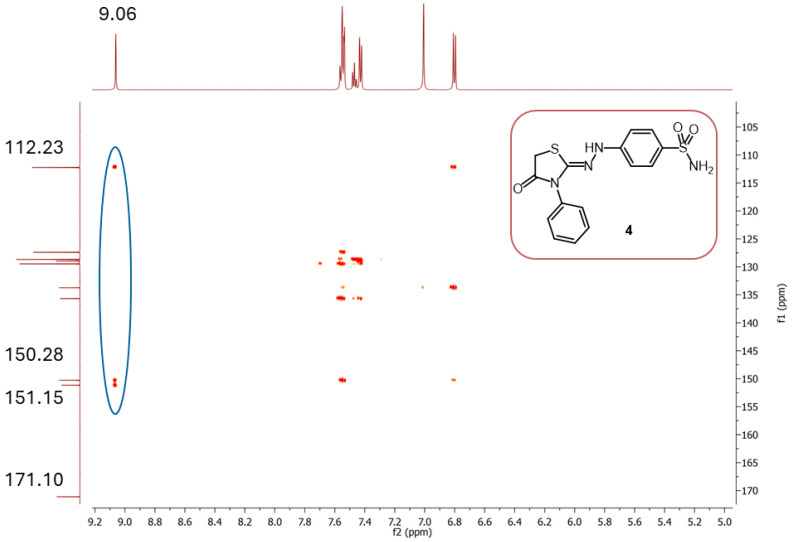
HMBC spectrum of compound **4**. In the horizontal axis, the ^1^H NMR spectrum is shown, and, in the vertical axis, the ^13^C NMR is shown. The significative coupling between NH with carbons is highlighted in blue.

**Figure 3:**
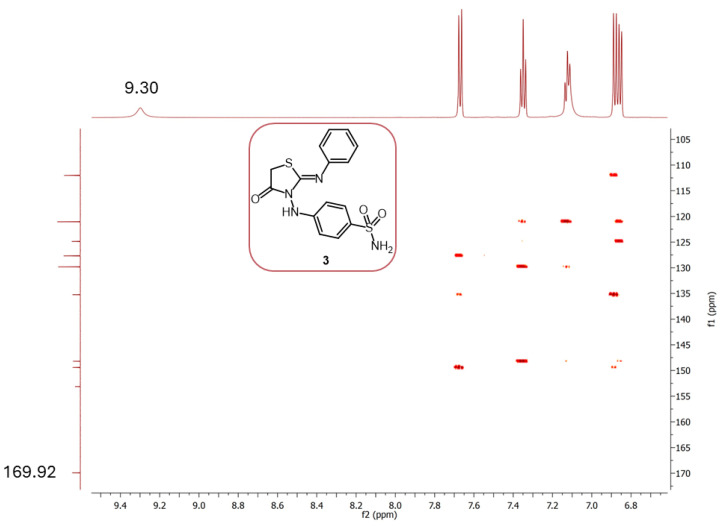
HMBC spectrum of compound **3**. In the horizontal axis, the ^1^H NMR spectrum is shown, and, in vertical axis, the ^13^C NMR is shown. No coupling was observed.

Indeed, ^1^H, ^13^C HMBC spectroscopy allows us to detect proton and carbon correlations over a range of 2–4 bonds. The ^1^H, ^13^C HMBC spectrum of compound **4** is shown in Figure 2. In this case, the benzene sulfonamide moiety binds the imine group, as demonstrated by the coupling of the NH proton with C2 and C2a. In contrast, for compound **3,** no coupling in the HMBC spectrum (Figure 3) was observed. The interaction of the NH proton with C2 and C4 could potentially have been observed in the HMBC spectrum. However, it was most likely undetected, due to the dihedral angle between the nuclei. The connection is most visible when the dihedral angle is 0° or 180°, and it is less noticeable if it is near to 90° [41]. Nevertheless, according to the obtained data, it is possible to assume that compound **3** is the regioisomer depicted in Table 1.

The most abundant regioisomer, regioisomer **3,** was used to proceed in the synthetic pathway and was condensed according to Knoevenagel conditions with differently substituted 2,3-indolinediones.

During this last step, a double bond was formed between thiazolidinone position 5 and position 3 of the indolinone nucleus. According to the literature, only the *Z* diastereoisomer was formed [42]. However, the configuration of the second double bond in position 2 of the thiazolidinone ring could not be determined, despite only one geometrical isomer being formed. Thus, for the molecular modeling investigation, both *Z* and *E* diastereoisomers were considered.

### 2.2. Carbonic Anhydrases Assay and Data Inhibition

Enzymatic assays were performed on isoforms I, II, IX, and XII to better understand how structural changes, specifically the natural and reciprocal position of the substituents on the thiazolidine ring positions 2 and 5, influenced the activity and selectivity of this series of molecules. The biological results are reported in Table 3.

The introduction of the phenyl ring in position 2 of the thiazolidinone ring resulted in the globally most potent derivatives toward isoforms hCA IX and hCA XII among the prior series of compounds [10]. Thus, while compound **5g** was found to be the most potent synthesized derivative against hCA IX (Ki = 2.5 nM), compound **5f** was the most potent toward hCA XII (Ki = 0.6 nM). However, although these derivatives are almost selective with respect to the hCA I isoform, they still retain activity toward the hCA II isozyme. For this series, small groups like methyl or fluorine at position 5 of the indolinone moiety improved the activity on isoform XII; in addition, this was observed for **5c**, **5f**, and **5h**. Moreover, compound **5e**, bearing methoxy moiety in position 5 of the indolinone ring, exhibited a potent inhibition toward hCA IX (Ki = 5.0 nM), but its activity toward hCA II was almost comparable (Ki = 9.6 nM). On the other hand, the presence of proton or fluorine at position 7, as in the cases of compounds **5g** and **5h**, improves the inhibition of isoform IX. These data indicate that, regarding hCA IX, the best inhibition results were achieved when no substituents were present on the indolinone ring, such as for compound **5g**. Nevertheless, the presence of a 5-methoxy group on the indolinone ring, such as for compound **5e,** also led to a potent inhibitor of hCA IX. Overall, the indolinone substitution effect on hCA IX inhibition potency could be summarized as follows: H > 5-OCH_3_ > 7F> 5-CH_3_ > 5-F > 5-NO_2_ > 7-Br > 5-Br > 5-Cl. When the inhibition of the hCA XII isoform was considered a different profile of the SARS, was observed being as follows: 5-CH_3_ > 7-F > 5-NO_2_ > H > 5-F > 5-OCH_3_ > 5-Cl > 7-Br > 5-Br.

### 2.3. Molecular Docking

Molecular docking experiments were conducted utilizing compound **5f**, one of the most promising molecules in the series, to estimate the theoretical binding affinity, rationalize the biological activity data, and guide future scaffold improvements. We applied our previously validated protocol [30] on hCA isoforms II, IX, and XII, considering the three-dimensional (3D) structures reported in the Protein Data Bank (PDB) repository, with PDB code 3F8E [43], 5FL4 [44], and 5MSA, respectivley [45].

Compound **5f** was selected to illustrate the putative binding mode due to its potent activity on both target isozymes (Ki hCA XII = 0.6 nM and Ki hCAIX = 14.9 nM)) and its promising selectivity index (SI hCA II/hCA IX = 4.75 and SI hCA II/hCA XII = 118).

**Figure 4:**
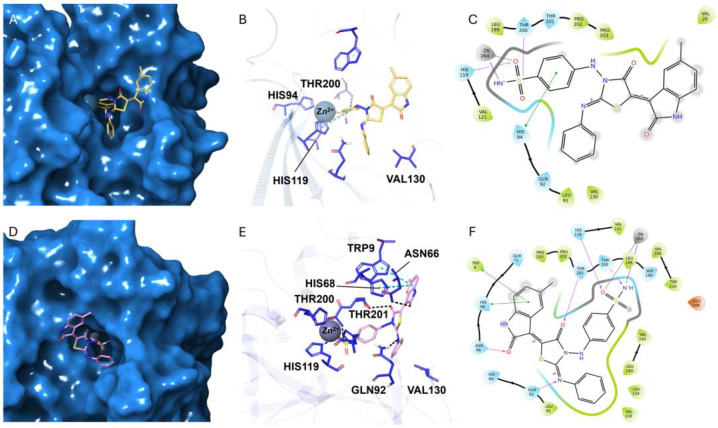
Putative binding mode of ***ZZ*-5f** (yellow sticks, panel (**A**)) and ***ZE*-5f** (pink sticks, panel (**D**)) on hCA IX (5FL4). Panels (**B**,**C**) show 3D and 2D interactions of compound ***ZZ*-5f** in the hCA IX catalytic site. Panels (**E**,**F**) show 3D and 2D interactions of compound ***ZE*-5f** in the hCA IX catalytic site.

The molecular docking experiments evidenced the ability of both *ZZ* and *ZE* diasteroisomers of compound **5f** to coordinate the zinc ion in the hCA IX and XII catalytic sites (Figure 4, panels A–F). Regarding the hCA IX–***ZZ-*5f** complex, this interaction is further stabilized by the formation of hydrogen bonds with His119 and Thr200, while His94 participates in p–p interactions, as depicted in Figure 4B,C. In the case of the ***ZE*-5f** stereoisomer (Figure 4F), interactions with His119, Thr200, Thr201, Gln92, Asn66, His68, and Trp9 stabilize its complex with hCA IX.

**Figure 5:**
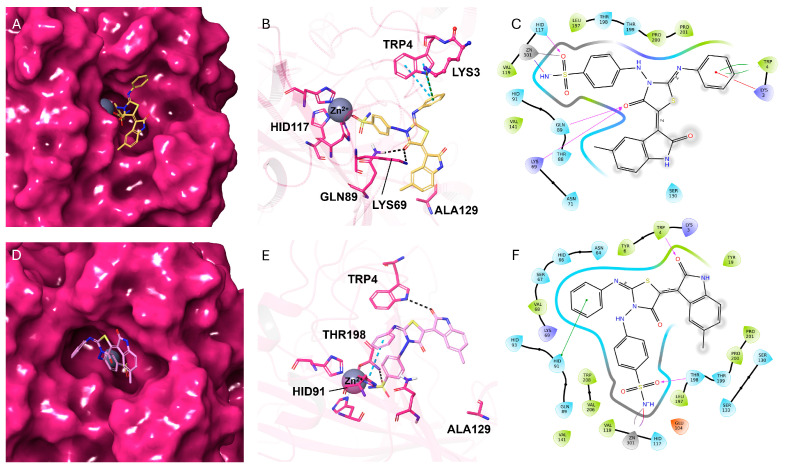
Putative binding mode of ***ZZ*-5f** (yellow sticks, panel (**A**)) and ***ZE*-5f** (pink sticks, panel (**D**)) on hCA XII (5MSA). Panels (**B**,**C**) show 3D and 2D interactions of compound ***ZZ*-5f** in the hCA XII catalytic site. Panels (**E**,**F**) show 3D and 2D interactions of compound ***ZE*-5f** in the hCA XI catalytic site.

Concerning the complex of compound **5f *ZZ*** and ***ZE*** stereoisomers with hCA XII, several interactions have been predicted (Figure 5, panels A–F). More in detail, in the case of compound ***ZZ*-5f** (Figure 5C), besides the coordination of the zinc ion, interactions with His117, Gln89, Lys69, Lys3, and Trp4 further stabilize the complex with hCA XII. Considering the ***ZE*-5f** diastereoisomer (Figure 5F), the coordination of the zinc ion was observed, as well as further stabilizing interactions with Thr198, His91, and Trp4.

**Figure 6:**
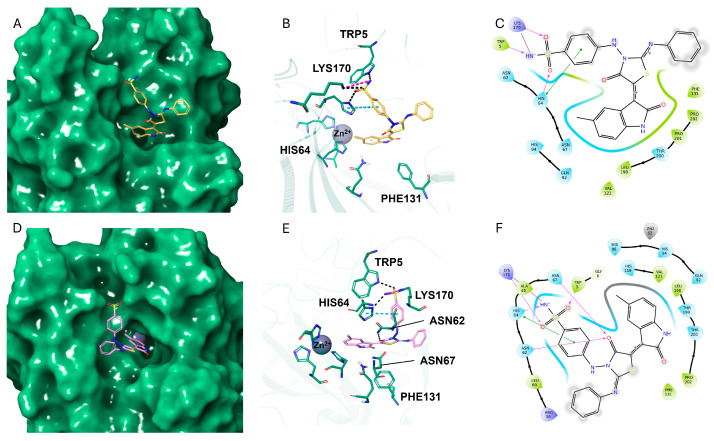
Putative binding mode of ***ZZ*-5f** (yellow sticks, panel (**A**)) and ***ZE*-5f** (pink sticks, panel (**D**)) on hCA II (3F8E). Panels (**B**,**C**) show 3D and 2D interactions of compound ***ZZ*-5f** in the hCA II catalytic site. Panels (**E**,**F**) show 3D and 2D interactions of compound ***ZE*-5f** in the hCA II catalytic site.

Conversely, when the predicted complexes between both ***ZZ*-5f** and ***ZE*-5f** with hCA II were examined, neither of the two diastereoisomers could coordinate the zinc ion with the sulfonamide group (Figure 6, panels C and F). This is likely due to the steric hindrance caused by Phe131, which prevents access to the binding cavity. This residue differs in isoforms IX and XII, which have Val130 and Ala129, respectively. However, an array of stabilizing interactions has been evidenced for both complexes (***ZZ*-5f**–hCA II and ***ZE*-5f**–hCA II). Compound ***ZZ*-5f** establishes interactions with Lys170, Trp5, and His64 with hCA II. Likewise, compound ***ZE*-5f** (Figure 6F) establishes interactions with Lys170, Ala65, His64, and Trp5 at the hCA II catalytic cavity entrance. Together, these data confirmed the biochemical activity.

In all probability, compound **5f** inhibits the hCA II isoform by impeding the access of the enzyme active site.

Molecular docking experiments allowed for a better understanding of the compounds’ putative binding modes. Moreover, they suggested that, although not capable of coordinating the zinc ion in the catalytic site, compound **5f** still retains residual activity toward the hCA II isozyme.

## 3. Materials and Methods

### 3.1. Chemistry

The starting materials, reagents, and solvents were purchased from Merk Life Science Milan, Italy, unless otherwise indicated, and used without further purification. Nuclear magnetic resonance (NMR) was recorded on a Bruker AMX 600 NMR spectrometer. ^1^H NMRand ^13^C NMRof **5a**–**i** compounds were measured in DMSO-*d*_6_ at 278.1 K temperature. Chemical shifts (δ) are indicated in parts per million (ppm), and the coupling constant (J) is expressed in hertz (Hz). Tetramethylsilane (TMS) was employed as an internal reference. TLC chromatography was performed using silica gel plates (Merck F 254, Darmstadt, Germany), and spots were visualized by UV light (254–366 nm). All melting points were calculated using the capillary method on a Stuart Scientific melting point instrument SMP30 (TEquipment, Long Branch, NJ, USA). The samples for MS analyses were solubilized in methanol (HPLC grade, with purity >99.9%). Positive and negative ESI-MS spectra were recorded with a high-resolution LTQ Orbitrap Elite™ mass spectrometer (Thermo Fisher Scientific, Waltham, MA, USA). The solutions were infused at a flow rate of 5.00 μL/min into the ESI source. The spectra were recorded in the range of *m*/*z* 100–2500, with a resolution of 120,000. The instrumental conditions were as follows: spray voltage, 3500 V; capillary temperature, 275 °C; sheath gas, 5–10 (arbitrary units); auxiliary gas, 3 (arbitrary units); sweep gas, 0 (arbitrary units); probe heater temperature, 50 °C. The compounds were named following IUPAC rules, as applied by ChemDraw 17.0 software.

All spectra are depicted in the Supplementary Materials.

#### 3.1.1. Synthetic Procedures

*Synthesis of 4-hydrazineylbenzenesulfonamide (*1*)*

The 4-Chlorobenzenesulfonamide (5.00 g; 0.026 mol) was suspended in 12 mL of hydrazine monohydrate (60/65%) in a microwave tube under stirring conditions. The reaction was performed in the microwave using reaction conditions of 250 psi for the pressure and 30 °C for the temperature for 40 min. After completion, the reaction was quenched by adding distilled water and stirring at 80 °C for 30 min. The solution was allowed to cool to rt and was stored in the fridge overnight. The formed pearl-white solid was filtered and dried in the oven at 50 °C and used without further purification. White solid; yield: 86.86%

*Synthesis of N-phenyl-2-(4-sulfamoylphenyl)hydrazine-1-carbothioamide (*2*)*

Compound **1** (2.00 g; 0.01 mol) was suspended in 10 mL of ethanol, and ‘*N*’-phenyl isothiocyanate (1.28 mL; 0.01 mol) was added dropwise. The reaction was stirred under reflux conditions for one night. The progression of the reaction was monitored though TLC using mobile phase CHCl_3_/IPA 10:1. The formation of the precipitate was observed, which was collected by filtration under vacuum conditions to obtain the desired product. White solid; yield: 96.23%; m.p. 199.8–200.8 °C; R*_f_* (DCM/MeOH 10:1) 0.29; ^1^H NMR (600 MHz, DMSO-*d*_6_) δ 6.83 (d, *J* = 8.8 Hz, 2H, Ar-H), 7.09 (s, 2H, -SO_2_NH_2_), 7.15 (t, *J* = 7.4 Hz, 1H, Ar-H), 7.31 (t, *J* = 7.8 Hz, 2H, Ar-H), 7.50 (d, *J* = 7.8 Hz, 1H, Ar-H), 7.68 (d, *J* = 8.6 Hz, 1H, Ar-H), 8.61 (s, 1H, -CSNHNH-Ar), 9.83 (s, 1H, Ar-NH), 9.88 (s, 1H, -CSNHNH-Ar); ^13^C NMR (151 MHz, DMSO-*d*_6_) δ 112.43 (2C, Ar-CH), 125.41 (1C, Ar-CH), 125.97 (1C, Ar-CH), 127.59 (2C, Ar-CH), 128.39 (1C, Ar-CH), 135.02 (1C, Ar-CNH), 139.60 (1C, Ar-CH), 151.32 (1C, Ar-CNNH), 181.79 (1C, -C=S).

*Synthesis of 4-((4-oxo-2-(phenylimino)thiazolidin-3-yl)amino)benzenesulfonamide (*3*) and 4-(2-(4-oxo-3-phenylthiazolidin-2-ylidene)hydrazineyl)benzenesulfonamide (*4*)*

To a suspension of compound 2 (3.0 g; 0.009 mol) and sodium acetate (4.60 g; 0.056 mol) in 25 mL of ethanol, ethyl bromoacetate (1.10 mL; 0.01 mol) was added dropwise. The reaction was left to react until completion under reflux monitoring with TLC using mobile phase CHCl_3_/IPA 10:1. The precipitated product was filtered under vacuum conditions to obtain regioisomer 3, and, after crystallization of mother liquor and filtration, the regioisomer 4 was obtained.

Compound **3**. Yield: 88.65%; m.p. 211–212 °C; R*_f_* (DCM/MeOH 10:1) 0.37; ^1^H NMR (600 MHz, DMSO-*d*_6_) δ 4.19 (s, 2H, -CH_2_), 6.85 (d, *J* = 8.0 Hz, 2H, Ar-H), 6.88 (d, *J* = 8.7 Hz, 2H, Ar-H), 7.05–7.19 (m, 3H, -SO_2_NH_2_ and Ar-H), 7.35 (t, *J* = 7.7 Hz, 2H, Ar-H), 7.67 (d, *J* = 8.6 Hz, 2H, Ar-H), 9.30 (s, 1H, -NNH); ^13^C NMR (151 MHz, DMSO-*d*_6_) δ 30.59 (1C, -CH_2_), 112.01 (2C, Ar-CH), 121.04 (1C,), 124.87 (1C, Ar-CH), 127.68 (2C, Ar-CH), 129.83 (1C, Ar-CH), 135.24 (1C, Ar-CSO_2_NH_2_), 148.20 (1C, Ar-CNHN), 149.42 (1C, -C=N), 153.13 (1C, Ar-CNH), 169.92 (1C, -C=O).

Compound **4**. Yield: 14.23%; m.p. 227–228° Ar-CH C; ^1^H NMR (600 MHz, DMSO-*d*_6_) δ 4.21 (s, 2H, -CH_2_), 6.80 (d, *J* = 8.7 Hz, 2H, Ar-H), 7.01 (s, 2H, -SO_2_NH_2_), 7.43 (d, *J* = 7.8 Hz, 2H, Ar-H), 7.46 (dd, *J* = 15.3, 7.8 Hz, 1H), Ar-H, 7.52–7.59 (m, 4H, Ar-H), 9.06 (s, 1H, -NNH); ^13^C NMR (151 MHz, DMSO-*d*_6_) δ 33.55 (1C, -CH_2_), 112.23 (2C, Ar-CH), 127.40 (2C, Ar-CH), 128.68 (1C, Ar-CH), 128.97 (1C, Ar-CH), 129.47 (1C, Ar-CH), 133.73 (1C, Ar-CNHCO), 135.67 (1C, Ar-CSO_2_NH_2_), 150.28 (1C, Ar-CNHN), 151.15 (1C, -C=N), 171.10 (1C, -C=O).

##### General Procedures for the Synthesis of **5a**–**i** Series

Final compounds **5a**–**i** were obtained according to Knoevenagel condensation using acid conditions. Compound **3** (1 eq) and sodium acetate (3 eq) were suspended in acetic acid. Then, the substituted isatin (1 eq) and the acetic anhydride (2 eq) were added. The reaction was stirred at 100 °C until completion, monitoring it through TLC with mobile phase CHCl_3_/IPA 10:1. The formation of a precipitate was observed, which was collected by vacuum filtration. The obtained solid was washed in water, filtered, and dried in the oven to obtain the final compounds.

*Synthesis of 4-((5-((Z)-5-chloro-2-oxoindolin-3-ylidene)-4-oxo-2-(phenylimino)thiazolidin-3-yl)amino)benzenesulfonamide (*5a*)*

Following the general procedure reported above, **5a** was synthesized from compound **3** (0.20 g; 0.0005 mol) and 5-chloro isatin (0.10 g; 0.0005 mol). An orange solid was obtained. Yield: 51.90%; m.p. 314.2–316.1 °C; R*_f_* (DCM/MeOH 10:1) 0.43; ^1^H NMR (600 MHz, DMSO-*d*_6_) δ 6.98 (d, *J* = 8.2 Hz, 3H, Ar-H and Ar-H of 5-chloro isatin), 7.01 (d, *J* = 8.8 Hz, 2H, Ar-H), 7.12 (d, *J* = 10.3 Hz, 2H, -SO_2_NH_2_), 7.22 (t, *J* = 7.4 Hz, 1H, Ar-H), 7.40–7.47 (m, 3H, Ar-H and Ar-H of 5-chloro isatin), 7.69 (d, *J* = 8.8 Hz, 2H, Ar-H), 8.82 (d, *J* = 2.1 Hz, 1H, Ar-H of 5-chloro isatin), 9.53 (s, 1H, -NNH), 11.37 (s, 1H, -CONH); ^13^C NMR (151 MHz, DMSO-*d*_6_) δ 112.34 (1C, Ar-CH), 112.38 (2C, Ar-CH), 121.22 (2C, Ar-CH), 121.86 (1C, -COC=CS), 125.04 (1C, -COC=CS), 125.59 (1C, Ar-CH), 126.33 (1C, Ar-CH), 127.57 (1C, Ar-CH), 127.77 (2C, Ar-CH), 130.02 (2C, Ar-CH), 131.33 (1C, Ar-CSO_2_NH_2_), 131.92 (1C, Ar-C-Cl), 135.64 (1C, Ar-CNHCO), 142.56 (1C, Ar-CNHN), 147.43 (1C, Ar-CN=C), 149.29 (1C, -SC=N), 150.39 (1C, Ar-CH), 164.03 (1C, -C=ONNH), 168.74 (1C, -C=ONH); ESI-HRMS (*m*/*z*) calculated for [M − H]^−^ ion species C_23_H_16_ClN_5_O_4_S_2_ = 524.0332, found 524.0220.

*4-((5-((Z)-5-nitro-2-oxoindolin-3-ylidene)-4-oxo-2-(phenylimino)thiazolidin-3-yl)amino)benzenesulfonamide (*5b*)*

Following the general procedure reported above, **5b** was synthesized from compound **3** (0.20 g; 0.0005 mol) and 5-nitro isatin (0.13 g; 0.001 mol). A yellow solid was obtained. Yield: 54.24%; m.p. 338.5–340 °C; *R_f_* (DCM/MeOH 10:1) 0.39; ^1^H NMR (600 MHz, DMSO-*d*_6_) δ 6.99 (d, *J* = 7.5 Hz, 2H, Ar-H), 7.03 (d, *J* = 8.8 Hz, 2H, Ar-H), 7.12 (s, 2H, -SO_2_NH_2_), 7.16 (d, *J* = 8.7 Hz, 1H, Ar-H of 5-nitro isatin), 7.24 (t, *J* = 7.4 Hz, 1H, Ar-H), 7.44 (t, *J* = 7.8 Hz, 2H, Ar-H), 7.70 (d, *J* = 8.8 Hz, 2H, Ar-H), 8.32 (dd, *J* = 8.7, 2.4 Hz, 1H, Ar-H of 5-nitro isatin), 9.59 (s, 1H, -NNH), 9.71 (d, *J* = 2.4 Hz, 1H, Ar-H of 5-nitro isatin), 11.93 (s, 1H, -CONH); ^13^C NMR (151 MHz, DMSO-*d*_6_) δ 111.19 (1C, Ar-CH), 112.46 (2C, Ar-CH), 120.61 (1C, Ar-CH), 121.21 (2C, Ar-CH), 123.50 (1C, -COC=CS), 124.17 (1C, -COC=CS), 125.72 (1C, Ar-C), 127.79 (2C, Ar-CH), 128.31 (1C, Ar-CH), 130.06 (2C, Ar-CH), 133.15 (1C, Ar-CSO_2_NH_2_), 135.73 (1C, Ar-CNHCO), 142.70 (1C, Ar-C-NO_2_), 147.33 (1C, Ar-CNHN), 149.06 (1C, Ar-CN=C), 149.27 (1C, -SC=N), 149.97 (1C, Ar-C), 164.00 (1C, -C=ONNH), 169.39 (1C, -C=ONH); ESI-HRMS (*m*/*z*) calculated for [M − H]^−^ ion species C_23_H_16_N_6_O_6_S_2_ = 535.0573, found 535.0463.

*4-((5-((Z)-5-fluoro-2-oxoindolin-3-ylidene)-4-oxo-2-(phenylimino)thiazolidin-3-yl)amino)benzenesulfonamide (*5c*)*

Following the general procedure described above, 5c was synthesized from compound 3 (0.20 g; 0.0005 mol) and 5-fluoro isatin (0.13 g; 0.001 mol). An orange solid was obtained. Yield: 35.71%; m.p. 297.5–299.8 °C; R*_f_* (DCM/MeOH 10:1) 0.37; ^1^H NMR (600 MHz, DMSO-*d*_6_) δ 6.94–6.99 (m, 3H, Ar-H and Ar-H of 5-fluoro isatin), 7.00 (d, *J* = 8.8 Hz, 2H, Ar-H), 7.12 (s, 2H, -SO_2_NH_2_), 7.22 (t, *J* = 7.4 Hz, 1H, Ar-H), 7.26 (td, *J* = 8.8, 2.7 Hz, 1H, Ar-H of 5-fluoro isatin), 7.43 (t, *J* = 7.8 Hz, 2H, Ar-H), 7.68 (d, *J* = 8.7 Hz, 2H, Ar-H), 8.58 (dd, *J* = 10.2, 2.7 Hz, 1H, Ar-H of 5-fluoro isatin), 9.53 (s, 1H, -NNH), 11.27 (s, 1H, -CONH); ^13^C NMR (151 MHz, DMSO-*d*_6_) δ 111.72 (d, ^3^*J_CF_* = 8.1 Hz, 1C, Ar-CH), 112.37 (2C, Ar-CH), 114.86 (d, ^2^*J_CF_* = 27.7 Hz, 1C, Ar-CH), 119.03 (d, ^2^*J_CF_* = 23.8 Hz, 1C, Ar-CH), 121.22 (2C, Ar-CH), 121.29 (1C, -COC=CS), 125.57 (1C, -COC=CS), 125.66 (d, ^4^*J_CF_* = 2.8 Hz, Ar-C), 127.77 (2C, Ar-CH), 130.01 (2C, Ar-CH), 131.07 (1C, Ar-CSO_2_NH_2_), 135.61 (1C, Ar-CNHCO), 140.28 (1C, Ar-C), 147.45 (1C, Ar-CNHN), 149.30 (1C, Ar-CN=C), 150.47 (1C, -SC=N), 157.98 (d, *^1^J_CF_* = 235.2 Hz, 1C, Ar-CF), 164.06 (1C, -C=ONNH), 168.96 (1C, -C=ONH); ESI-HRMS (*m*/*z*) calculated for [M − H]^−^ ion species C_23_H_16_FN_5_O_4_S_2_ = 508.0628, found 508.0523.

*4-((5-((Z)-5-bromo-2-oxoindolin-3-ylidene)-4-oxo-2-(phenylimino)thiazolidin-3-yl)amino)benzenesulfonamide (*5d*)*

Following the general procedure shown above, 5d was synthesized from compound 3 (0.20 g; 0.0005 mol) and 5-bromo isatin (0.12 g; 0.0005 mol). An orange solid was obtained. Yield: 60.51%; m.p. 331.4–332.7 °C; R*_f_* (DCM/MeOH 10:1) 0.39; ^1^H NMR (600 MHz, DMSO-*d*_6_) δ 6.98 (d, *J* = 8.3 Hz, 1H, Ar-H of 5-bromo isatin), 7.02 (d, *J* = 7.7 Hz, 2H, Ar-H), 7.06 (d, *J* = 8.7 Hz, 2H, Ar-H), 7.18 (s, 2H, -SO_2_NH_2_), 7.27 (t, *J* = 7.4 Hz, 1H, Ar-H), 7.48 (t, *J* = 7.8 Hz, 2H, Ar-H), 7.61 (dd, *J* = 8.3, 1.8 Hz, 1H, Ar-H of 5-bromo isatin), 7.74 (d, *J* = 8.7 Hz, 2H, Ar-H), 9.01 (d, *J* = 1.6 Hz, 1H, Ar-H of 5-bromo isatin), 9.58 (s, 1H, -NNH), 11.43 (s, 1H, -CONH); ^13^C NMR (151 MHz, DMSO-*d*_6_) δ 112.39 (2C, Ar-CH), 112.82 (1C, Ar-CH), 114.03 (1C, Ar-CH), 121.22 (2C, Ar-CH), 122.33 (1C, Ar-CBr), 124.90 (1C, -COC=CS), 125.59 (1C, -COC=CS), 127.78 (2C, Ar-CH), 130.02 (2C, Ar-CH), 130.35 (1C, Ar-CSO_2_NH_2_), 131.33 (1C, Ar-CH), 134.70 (1C, Ar-C), 135.64 (1C, Ar-CNHCO), 142.91 (1C, Ar-C), 147.43 (1C, Ar-CNHN), 149.30 (1C, Ar-CN=C), 150.39 (1C, -SC=N), 164.04 (1C, -C=ONNH), 168.62 (1C, -C=ONH); ESI-HRMS (*m*/*z*) calculated for [M − H]^−^ ion species C_23_H_16_BrN_5_O_4_S_2_ = 567.9827, found 567.9724.

*4-((5-((Z)-5-methoxy-2-oxoindolin-3-ylidene)-4-oxo-2-(phenylimino)thiazolidin-3-yl)amino)benzenesulfonamide (*5e*)*

Following the general procedure shown above, 5e was synthesized from compound 3 (0.20 g; 0.0005 mol) and 5-methoxy isatin (97 mg; 0.0005 mol). An orange solid was obtained. Yield: 45.30%; m.p. 328.8–330.1 °C; R*_f_* (DCM/MeOH 10:1) 0.37; ^1^H NMR (600 MHz, DMSO-*d*_6_) δ 3.74 (s, 3H, -OCH_3_), 6.87 (d, *J* = 8.5 Hz, 1H, Ar-H of 5-methoxy isatin), 6.97 (d, *J* = 7.6 Hz, 2H, Ar-H), 6.99–7.02 (m, 3H, Ar-H and Ar-H of 5-methoxy isatin), 7.13 (s, 2H, -SO_2_NH_2_), 7.21 (t, *J* = 7.4 Hz, 1H, Ar-H), 7.42 (t, *J* = 7.8 Hz, 2H, Ar-H), 7.69 (d, *J* = 8.7 Hz, 2H, Ar-H), 8.49 (d, *J* = 2.5 Hz, 1H, Ar-H of 5-methoxy isatin), 9.53 (s, 1H, -NNH), 11.04 (s, 1H, -CONH); ^13^C NMR (151 MHz, DMSO-*d*_6_) δ 56.00 (1C, -OCH_3_), 111.35 (1C, Ar-CH), 112.31 (2C, Ar-CH), 113.72 (1C, Ar-CH), 118.77 (1C, Ar-CH), 121.14 (1C, Ar-CH), 121.24 (2C, Ar-CH), 125.46 (1C, -COC=CS), 126.76 (1C, -COC=CS), 127.79 (2C, Ar-CH), 129.34 (1C, Ar-CSO_2_NH_2_), 129.98 (2C, Ar-CH), 135.55 (1C, Ar-C), 137.78 (1C, Ar-CNHCO), 147.56 (1C, Ar-CNHN), 149.34 (1C, Ar-CN=C), 150.75 (1C, -SC=N), 155.09 (1C, Ar-C-OCH_3_), 164.09 (1C, -C=ONNH), 168.95 (1C, -C=ONH); ESI-HRMS (*m*/*z*) calculated for [M − H]^−^ ion species C_24_H_19_N_5_O_5_S_2_ = 520.0828, found 520.0725.

*4-((5-((Z)-5-methyl-2-oxoindolin-3-ylidene)-4-oxo-2-(phenylimino)thiazolidin-3-yl)amino)benzenesulfonamide (*5f*)*

Following the general procedure shown above, 5f was synthesized from compound 3 (0.20 g; 0.0005 mol) and 5-methyl isatin (88 mg; 0.0005 mol). An orange solid was obtained. Yield: 40.65%; m.p. 333.9–334.6 °C; R*_f_* (DCM/MeOH 10:1) 0.39; ^1^H NMR (600 MHz, DMSO-*d*_6_) δ 2.29 (s, 3H, -CH_3_), 6.84 (d, *J* = 7.9 Hz, 1H, Ar-H of 5-methyl isatin), 6.96–7.01 (m, 4H, Ar-H and Ar-H of 5-methyl isatin), 7.13 (s, 2H, -SO_2_NH_2_), 7.21 (t, *J* = 7.5 Hz, 2H, Ar-H), 7.42 (t, *J* = 7.8 Hz, 2H, Ar-H), 7.69 (d, *J* = 8.8 Hz, 2H, Ar-H), 8.63 (s, 1H, Ar-H of 5-methyl isatin), 9.51 (s, 1H, -NNH), 11.12 (s, 1H, -CONH); ^13^C NMR (151 MHz, DMSO-*d*_6_) δ 21.35 (1C, -CH_3_), 110.62 (1C, Ar-CH), 112.29 (2C, Ar-CH), 120.65 (1C, Ar-CH), 121.25 (2C, Ar-CH), 125.43 (1C, -COC=CS), 126.56 (1C, -COC=CS), 127.80 (2C, Ar-CH), 128.65 (1C, Ar-CH), 128.73 (1C, Ar-CH), 129.98 (2C, Ar-CH), 131.24 (1C, -CSO_2_NH_2_), 133.16 (1C, Ar-C-CH_3_), 135.55 (1C, Ar-CNHCO), 141.73 (1C, Ar-C), 147.61 (1C, Ar-CNHN), 149.37 (1C, Ar-CN=C), 150.83 (1C, -SC=N), 163.97 (1C, -C=ONNH), 169.00 (1C, -C=ONH); ESI-HRMS (*m*/*z*) calculated for [M − H]^−^ ion species C_24_H_19_N_5_O_4_S_2_ = 504.0878, found 504.0784.

*4-((4-oxo-5-((Z)-2-oxoindolin-3-ylidene)-2-(phenylimino)thiazolidin-3-yl)amino)benzenesulfonamide (*5g*)*

Following the general procedure shown above, 5g was synthesized from compound 3 (0.20 g; 0.0005 mol) and isatin (81 mg; 0.0005 mol). An orange/red solid was obtained. Yield: 45.93%; m.p. 326–327 °C; R*_f_* (DCM/MeOH 10:1) 0.39; ^1^H NMR (600 MHz, DMSO-*d*_6_) δ 6.96 (d, *J* = 7.9 Hz, 1H, Ar-H of isatin), 6.98 (t, *J* = 8.6 Hz, 4H, Ar-H), 7.06 (t, *J* = 7.8 Hz, 1H, Ar-H), 7.12 (s, 2H, -SO_2_NH_2_), 7.21 (t, *J* = 7.4 Hz, 1H, Ar-H of isatin), 7.39 (t, *J* = 7.7 Hz, 1H, Ar-H of isatin), 7.42 (t, *J* = 7.8 Hz, 2H, Ar-H), 7.68 (d, *J* = 8.8 Hz, 2H, Ar-H), 8.78 (d, *J* = 7.9 Hz, 1H, Ar-H of isatin), 9.51 (s, 1H, -NNH), 11.23 (s, 1H, -CONH); ^13^C NMR (151 MHz, DMSO-*d*_6_) δ 110.93 (1C, Ar-CH), 112.32 (2C, Ar-CH), 120.59 (1C, Ar-C), 121.25 (2C, Ar-CH), 122.50 (1C, Ar-CH), 125.45 (1C, -COC=CS), 126.30 (1C, -COC=CS), 127.78 (2C, Ar-CH), 128.32 (1C, Ar-CH), 129.08 (1C, -CSO_2_NH_2_), 129.98 (2C Ar-CH), 132.72 (1C, Ar-C), 135.54 (1C, Ar-CNHCO), 143.93 (1C, Ar-C), 147.59 (1C, Ar-CNHN), 149.38 (1C, Ar-CN=C), 150.77 (1C, -SC=N), 163.97 (1C, -C=ONNH), 168.96 (1C, -C=ONH); ESI-HRMS (*m*/*z*) calculated for [M − H]^−^ ion species C_23_H_17_N_5_O_4_S_2_ = 490.0722, found 490.0628.

*4-((5-((Z)-7-fluoro-2-oxoindolin-3-ylidene)-4-oxo-2-(phenylimino)thiazolidin-3-yl)amino)benzenesulfonamide (*5h*)*

Following the general procedure shown above, 5h was synthesized from compound 4 (0.20 g; 0.0005 mol) and 7-fluoro isatin (90 mg; 0.0005 mol). An orange/red solid was obtained. Yield: 35.71%; m.p. 336.1–336.6 °C; R*_f_* (DCM/MeOH 10:1) 0.40; ^1^H NMR (600 MHz, DMSO-*d*_6_) δ 7.05 (d, *J* = 7.5 Hz, 2H, Ar-H), 7.07 (d, *J* = 8.8 Hz, 2H, Ar-H), 7.14 (td, *J* = 8.2, 5.2 Hz, 1H, Ar-H of 7-fluoro isatin), 7.19 (s, 2H, -SO_2_NH_2_), 7.29 (t, *J* = 7.4 Hz, 1H, Ar-H), 7.40 (t, *J* = 9.1 Hz, 1H, Ar-H of 7-fluoro isatin), 7.50 (t, *J* = 7.8 Hz, 2H, Ar-H), 7.75 (d, *J* = 8.8 Hz, 2H, Ar-H), 8.71 (d, *J* = 8.0 Hz, 1H, Ar-H of 7-fluoro isatin), 9.59 (s, 1H, -NNH), 11.83 (s, 1H, -CONH); ^13^C NMR (151 MHz, DMSO-*d*_6_) δ 112.37 (2C, Ar-CH), 119.11 (d, *^2^J_CF_* = 16.9 Hz, 1C, Ar-CH), 121.23 (2C, Ar-CH), 123.01 (d, *^3^J_CF_* = 5.9 Hz, Ar-CH), 123.27 (d, *^4^J_CF_* = 4.3 Hz), 124.31 (d, *^4^J_CF_* = 2.8 Hz, Ar-CH), 125.36 (d, *^4^J_CF_* = 4.3 Hz, -COC=CS), 125.55 (1C, -COC=CS), 127.77 (2C, Ar-CH), 130.02 (2C, Ar-CH), 130.94 (d, *^2^J_CF_* = 13.3 Hz, Ar-CNHCO), 131.26 (1C, -CSO_2_NH_2_), 135.59 (1C, Ar-C), 147.10 (d, *^1^J_CF_* = 242.4 Hz, Ar-CF), 147.48 (1C, Ar-CNHN), 149.34 (1C, Ar-CN=C), 150.40 (1C, -SC=N), 163.80 (1C, -C=ONNH), 168.80 (1C, -C=ONH); ESI-HRMS (*m*/*z*) calculated for [M − H]^−^ ion species C_23_H_16_FN_5_O_4_S_2_ = 508.0628, found 508.0527.

*4-((5-((Z)-7-bromo-2-oxoindolin-3-ylidene)-4-oxo-2-(phenylimino)thiazolidin-3-yl)amino)benzenesulfonamide (*5i*)v*

Following the general procedure shown above, 5i was synthesized from compound 4 (0.20 g; 0.0005 mol) and 7-bromo isatin (0.12 g; 0.0005 mol). An orange/red solid was obtained. Yield: 51.91%; m.p. 344.9–346.0 °C; R*_f_* (DCM/MeOH 10:1) 0.41; ^1^H NMR (600 MHz, DMSO-*d*_6_) δ 6.98 (d, *J* = 7.5 Hz, 2H, Ar-H), 7.00 (d, *J* = 8.8 Hz, 2H, Ar-H), 7.03 (t, *J* = 8.1 Hz, 1H, Ar-H of 7-bromo isatin), 7.12 (s, 2H, -SO_2_NH_2_), 7.22 (t, *J* = 7.4 Hz, 1H), 7.43 (t, *J* = 7.8 Hz, 2H, Ar-H), 7.59 (d, *J* = 8.1 Hz, 1H, Ar-H of 7-bromo isatin), 7.68 (d, *J* = 8.8 Hz, 2H, Ar-H), 8.82 (d, *J* = 7.9 Hz, 1H, Ar-H of 7-bromo isatin), 9.52 (s, 1H, -NNH), 11.52 (s, 1H, -CONH); ^13^C NMR (151 MHz, DMSO-*d*_6_) δ 103.24 (1C, Ar-CBr), 112.37 (2C, Ar-CH), 121.23 (2C, Ar-CH), 122.29 (1C, Ar-CH), 123.95 (1C, Ar-CH), 125.56 (1C, Ar-CH), 125.67 (1C, COC=CS), 127.19 (1C, -COC=CS), 127.76 (2C, Ar-CH), 130.02 (2C, Ar-CH), 131.43 (1C, -CSO_2_NH_2_), 135.00 (1C, Ar-C), 135.59 (1C Ar-C), 142.85 (1C, Ar-CH), 147.48 (1C, Ar-CNHN), 149.33 (1C, Ar-CN=C), 150.42 (1C, -SC=N), 163.80 (1C, -C=ONNH), 168.88 (1C, -C=ONH); ESI-HRMS (*m*/*z*) calculated for [M − H]^−^ ion species C_23_H_16_BrN_5_O_4_S_2_ = 567.9827, found 567.9738.

### 3.2. Biochemical Evaluation of hCA Inhibition

A stopped-flow instrument was used, according to the previously reported methodology, to measure the CA (carbonic anhydrase)-catalyzed CO_2_ hydration/inhibition [46]. For 10 to 100 s, the CA-catalyzed CO_2_ hydration reaction’s initial rates were observed. To calculate the inhibition constants, the CO_2_ concentrations ranged from 1.7 to 17 mM. From the total recorded rates, the uncatalyzed rates were removed. Stock solutions of inhibitors (10 mM) and dilutions up to 0.01 nM were prepared in distilled-deionized water. Before the experiment started, the inhibitor and enzyme solutions were preincubated for 15 min at room temperature to allow for the formation of the enzyme/inhibitor (E/I) complex. The inhibition constants were obtained by non-linear least-squares methods using PRISM 3 software, as reported earlier, and represent the mean from at least three different determinations. hCA I, hCA II, hCA IX, and hCA XII (catalytic domain) were recombinant proteins produced in-house using our standardized protocol, and their concentration in the assay system was in the range of 3–10 nM. AAZ (acetazolamide) was used as a reference carbonic anhydrase inhibitor (CAI) [47,48,49].

### 3.3. Molecular Modeling

Molecular docking experiments have been carried out to predict the possible binding mode of **5a**–**i** series on hCA IX and XII and hCA II isoforms.

Maestro GUI [50] was used to build the three-dimensional compounds structure. The most stable conformation of ligands was established by molecular mechanics conformational analysis performed applying MacroModel software version 9.2 [51] and considering Molecular Force Fields (MMFFs) [52] in water solution, allowing maximum 5000-step Monte Carlo analysis and a convergence criterion of 0.05 kcal/mol.

The hCA II, IX, and XII crystal structures were downloaded from the RCSB Protein Data Bank [53] and have the following PDB codes: 3F8E [43], 5FL4 [17], and 5MSA, respectivley [45]. These were selected from the available ones due to their higher resolution. Furthermore, the alignment with the other 3D structure showed no appreciable differences that would justify the application of ensemble docking. The protein optimization was carried out employing Maestro Protein Preparation Wizard, leaving the default settings. The previously validated Quantum-Mechanics-Polarized Ligand (QMPL) Docking protocol has been applied [54,55].

## 4. Conclusions

Based on previous results, a small library of 4-((4-oxo-5-((*Z*)-2-oxoindolin-3-ylidene)-2-(phenylimino)thiazolidin-3-yl)amino)benzenesulfonamide has been designed and synthesized by hybridizing highly represented scaffolds in anticancer-related drugs. The presence of the zinc-binding group benzenesulfonamide was combined with a disubstituted thiazolidinone ring condensed with different indolinones moieties to identify the most promising structural features for the selective inhibition of tumor-associated hCA IX and XII isoforms. In particular, with respect to the previously reported compounds, both the nature and the substitution pattern on the thiazolidinone central core was modified. During the synthetic process, the formation of two regioisomers (**3** and **4**) was observed. The regioisomers were characterized by means of ^1^H, ^13^C HMBC NMR spectroscopic experiments. All compounds were also submitted to biochemical evaluation in order to assess their activity and selectivity toward four isoforms of hCA, namely isozymes hCA I, hCA II, hCA IX, and hCA XII. The data confirmed that all of the compounds exhibited a selective inhibition of hCAIX and hCA XII, with respect to isozyme hCA I. Nevertheless, the activity toward the hCA II isoform is retained in many of the compounds, although it is most probably related to the obstruction of the catalytic cavity rather than to an interaction of the benzenesulfonamide with the zinc ion. This indication was corroborated by molecular docking experiments, which indicated the putative binding mode of compound **5f** in hCA II, hCA IX, and hCA XII. Altogether, this information indicates that derivatives **5a**–**i** exhibited some particular features such as the presence of a bulky portion constituted by the phenylimino and the indolinone group, respectively, in position 2 and 5 of the thiazolidinone ring, which prevent the orientation of the zinc-binding group toward the zinc in the case of hCA II. Compounds **5a**–**i** are generally more active toward the target isozymes IX and XII, but further optimization is required to gain full selectivity.

## Figures and Tables

**Table 1:** Registered ^13^C NMR chemical shifts for compound **3** (solvent DMSO-*d*_6_).

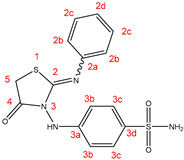											
C	5	3b	2b	2d	3c	2c	3d	3a	2	2a	4
δ	30.59	112.01	121.04	124.87	127.68	129.83	135.24	148.20	149.42	153.13	169.92

**Table 2:** Registered ^13^C NMR chemical shifts for compound **4** (solvent DMSO-*d*_6_).

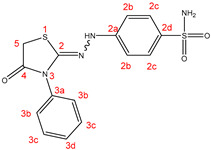											
C	5	2b	2c	3b	3d	3c	3a	2d	2a	2	4
δ	33.55	112.23	127.4	128.68	128.97	129.47	133.73	135.67	150.28	151.15	171.10

**Table 3:** Inhibition data of **5a**–**i** series toward hCA isozymes.

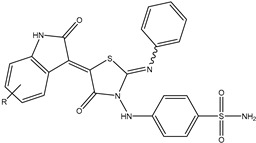					
Ki nM					
Code	R	hCA I	hCA II	hCA IX	hCA XII
**5a**	5-Cl	4591	245.8	99.4	61.1
**5b**	5-NO_2_	6031	159.5	34.6	7.5
**5c**	5-F	5880	6.9	23.1	21.8
**5d**	5-Br	>10,000	104.3	87.4	193.0
**5e**	5-OCH_3_	9064	9.6	5.0	53.7
**5f**	5-CH_3_	>10,000	70.8	14.9	0.6
**5g**	-H	5278	9.6	2.5	16.8
**5h**	7-F	6625	4.1	12.7	1.3
**5i**	7-Br	7685	17.8	50.3	94.0
**AAZ**		250.0	12.0	25.0	5.7

## Data Availability

Data are contained within the article and Supplementary Materials.

## Supplementary Materials

The following supporting information can be downloaded at: https://www.mdpi.com/article/10.3390/molecules29184444/s1, ^1^H NMR, ^13^C NMR, and ESI-HRMS spectra of **5a**–**i** compounds.
