# Supplementary material for: Privileged Scaffold Hybridization in the Design of Carbonic Anhydrase Inhibitors
Source: Molecules. 2024 Sep 19;29(18):4444. doi: 10.3390/molecules29184444 (PMC11433937; doi:10.3390/molecules29184444)

Supplementary Materials

Privileged scaffolds hybridization in the design of carbonic anhydrase inhibitors

Daniela Secci [1], Erica Sanna [1], Simona Distinto [1] Alessia Onali [1], Antonio Lupia [1-2], Laura Demuru [1], Giulia Atzeni [1], Rita Meleddu [1*], Filippo Cottiglia [1], Andrea Angeli [3], Claudiu T. Supuran [3*] and Elias Maccioni [1]

[1]Department of Life and Environmental Sciences, University of Cagliari, Cittadella Universitaria di Monserrato, Monserrato, 09042 Cagliari, Italy
[2]Net4Science S.r.l, Università degli Studi "Magna Græcia" di Catanzaro, Catanzaro, 88100, Italy
[3] Dipartimento NEUROFARBA, Sezione di Scienze Farmaceutiche, Università degli Studi di Firenze, Sesto Fiorentino, Florence, Italy.
[*]Correspondence: R.Meleddu, rita.meleddu@unica.it; C.T.Supuran, claudiu.supuran@unifi.it

Table of contents

^{1}H NMR and ^{13}C NMR of **2-5a-i** compounds 3
^{1}H NMR and ^{13}C NMR of compound **2** 3
^{1}H NMR and ^{13}C NMR of compound **3** 4
^{1}H NMR and ^{13}C NMR of compound **4** 5
^{1}H NMR and ^{13}C NMR of compound **5a** 6
^{1}H NMR and ^{13}C NMR of compound **5b** 7
^{1}H NMR and ^{13}C NMR of compound **5c** 8
^{1}H NMR and ^{13}C NMR of compound **5d** 9
^{1}H NMR and ^{13}C NMR of compound **5e** 10
^{1}H NMR and ^{13}C NMR of compound **5f** 11
^{1}H NMR and ^{13}C NMR of compound **5g** 12
^{1}H NMR and ^{13}C NMR of compound **5h** 13
^{1}H NMR and ^{13}CNMR of compound **5i** 14
ESI-HRMS spectra of **5a-i** compounds ($[M-H]^-$) 15
MS spectrum of compound **5a** 15
MS spectrum of compound **5b** 15
MS spectrum of compound **5c** 16
MS spectrum of compound **5d** 16
MS spectrum of compound **5e** 17
MS spectrum of compound **5f** 17
MS spectrum of compound **5g** 18

MS spectrum of compound **5h** 18
MS spectrum of compound **5i** 19

^{1}H NMR and ^{13}C NMR of **2-5a-i** compounds

^{1}H NMR and ^{13}C NMR of compound **2**

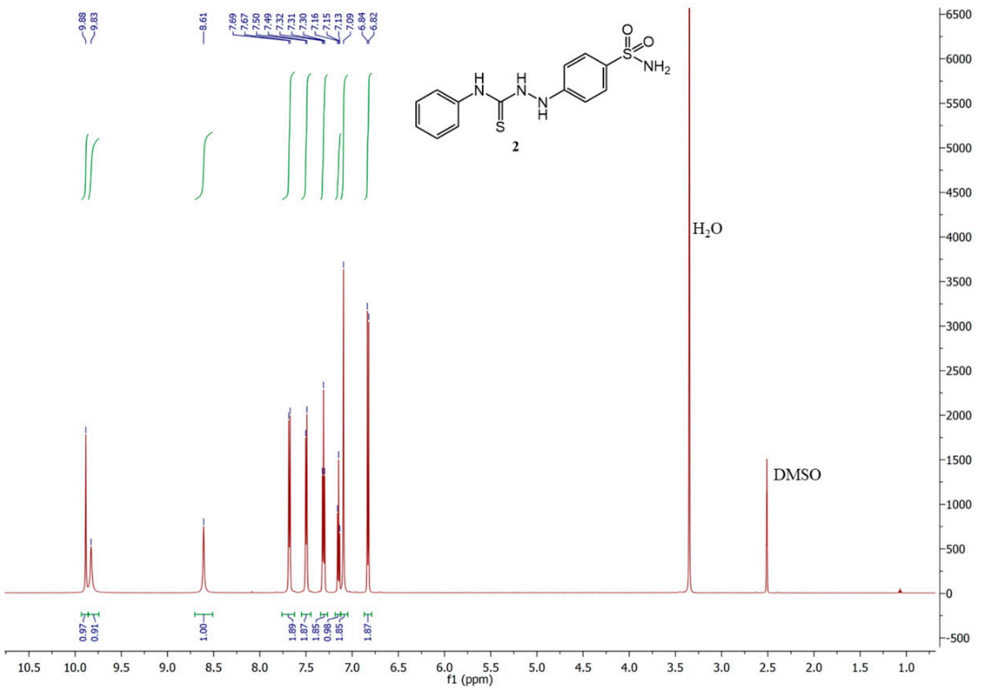

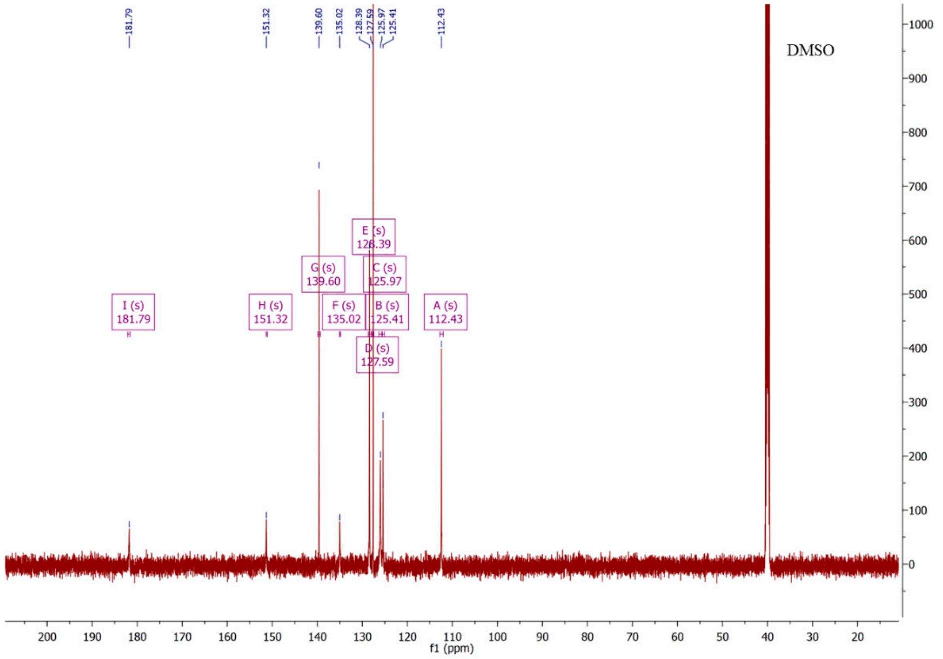

^{1}H NMR and ^{13}C NMR of compound **3**

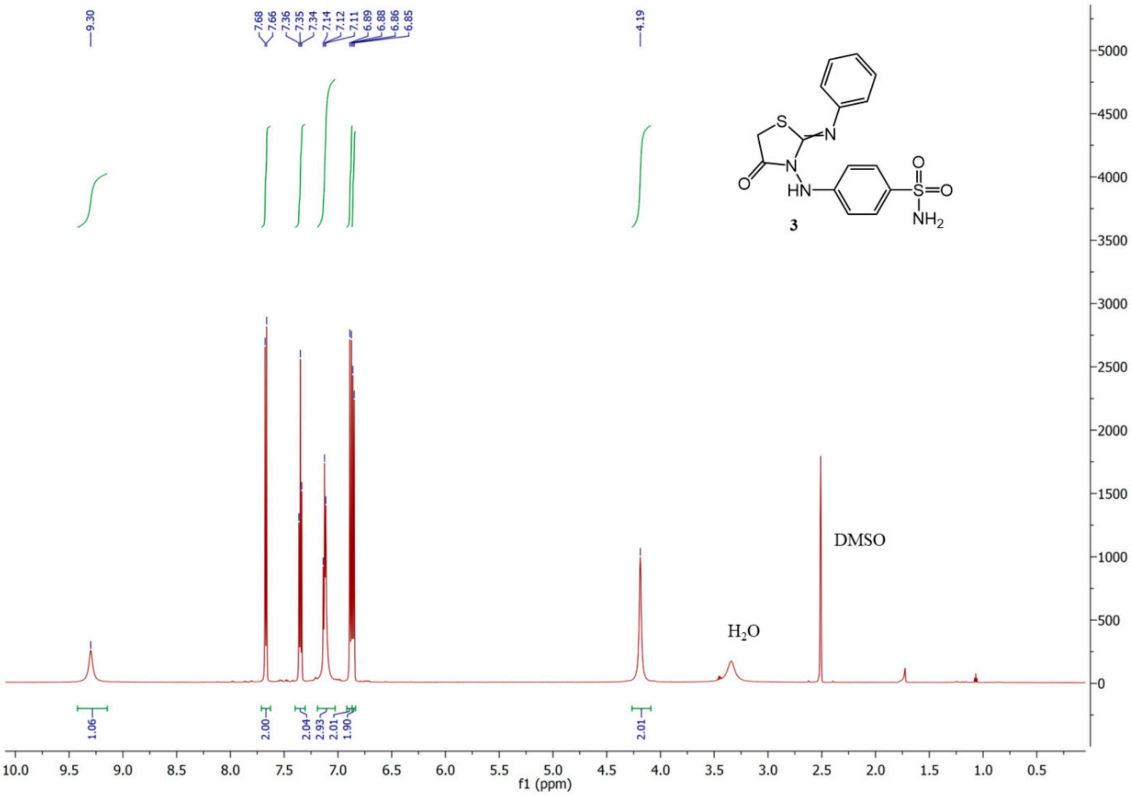

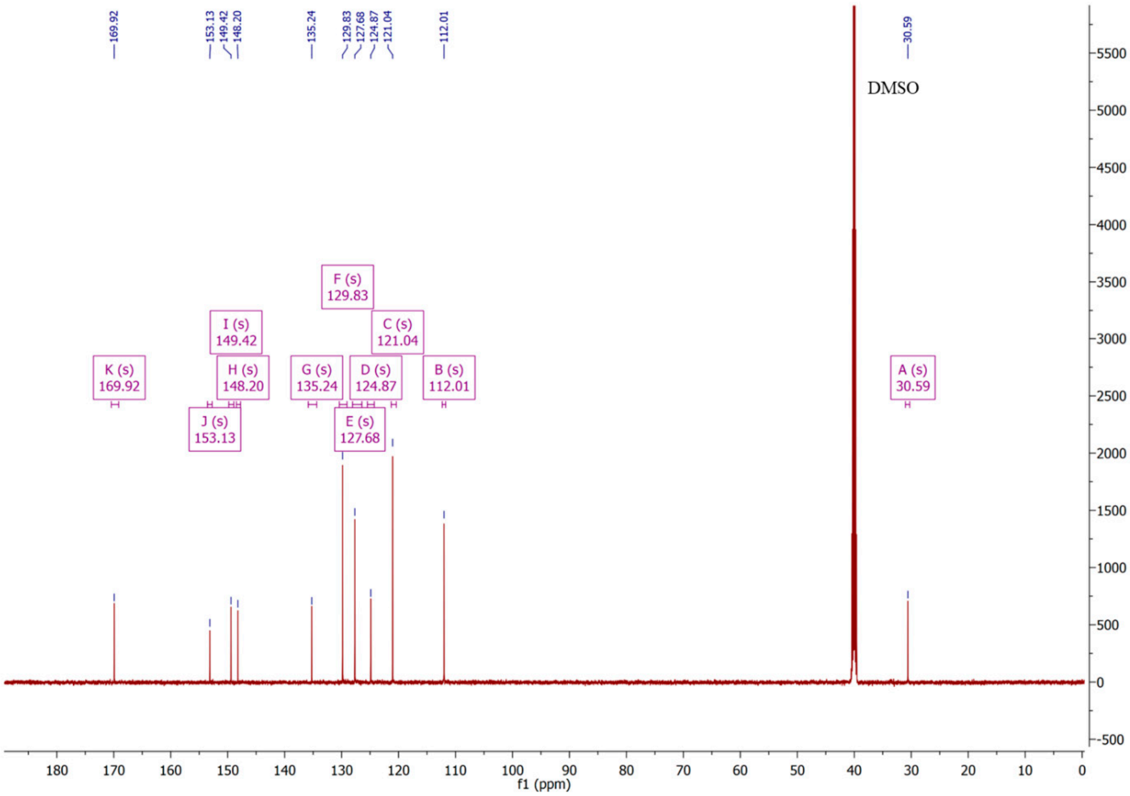

^{1}H NMR and ^{13}C NMR of compound **4**

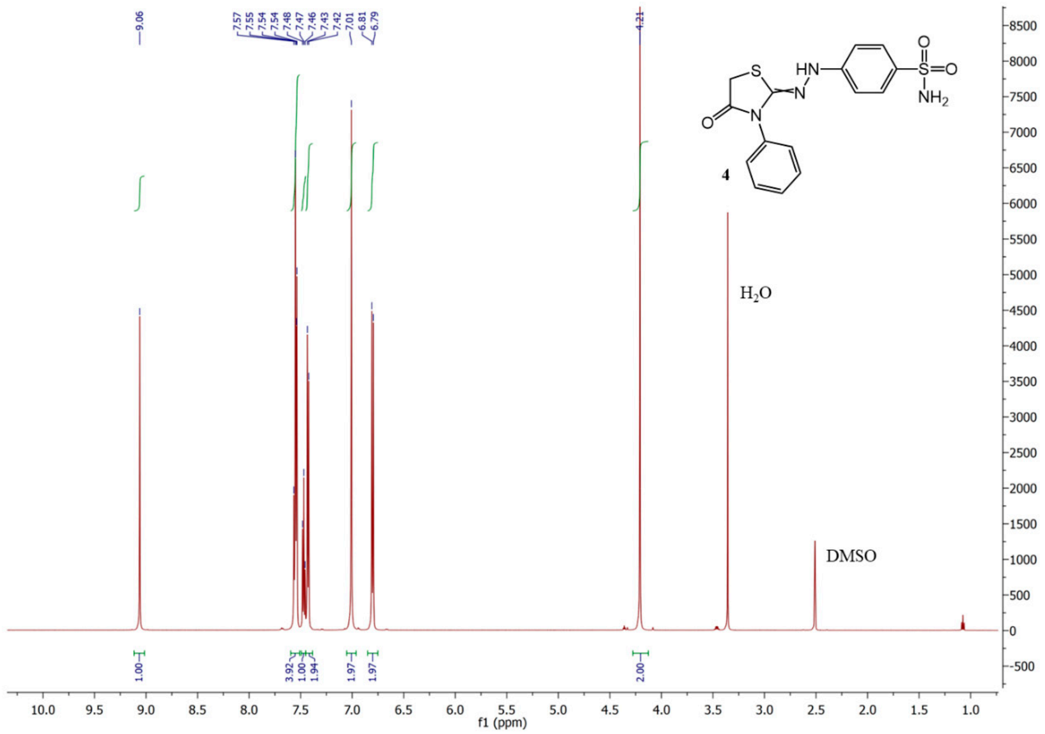

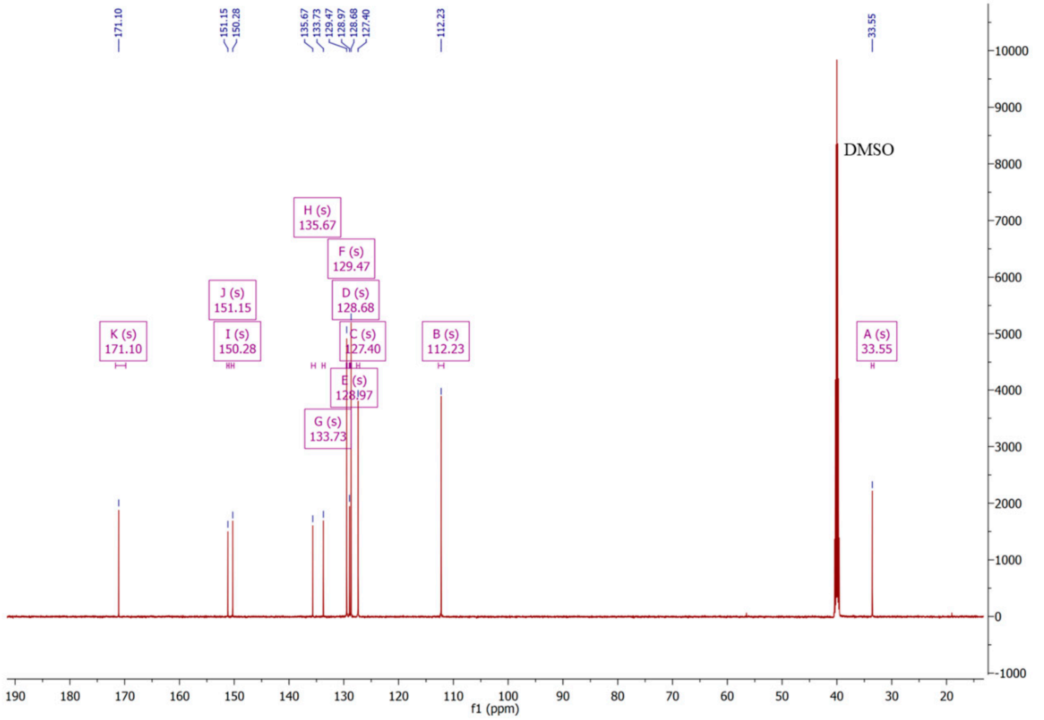

^{1}H NMR and ^{13}C NMR of compound **5a**

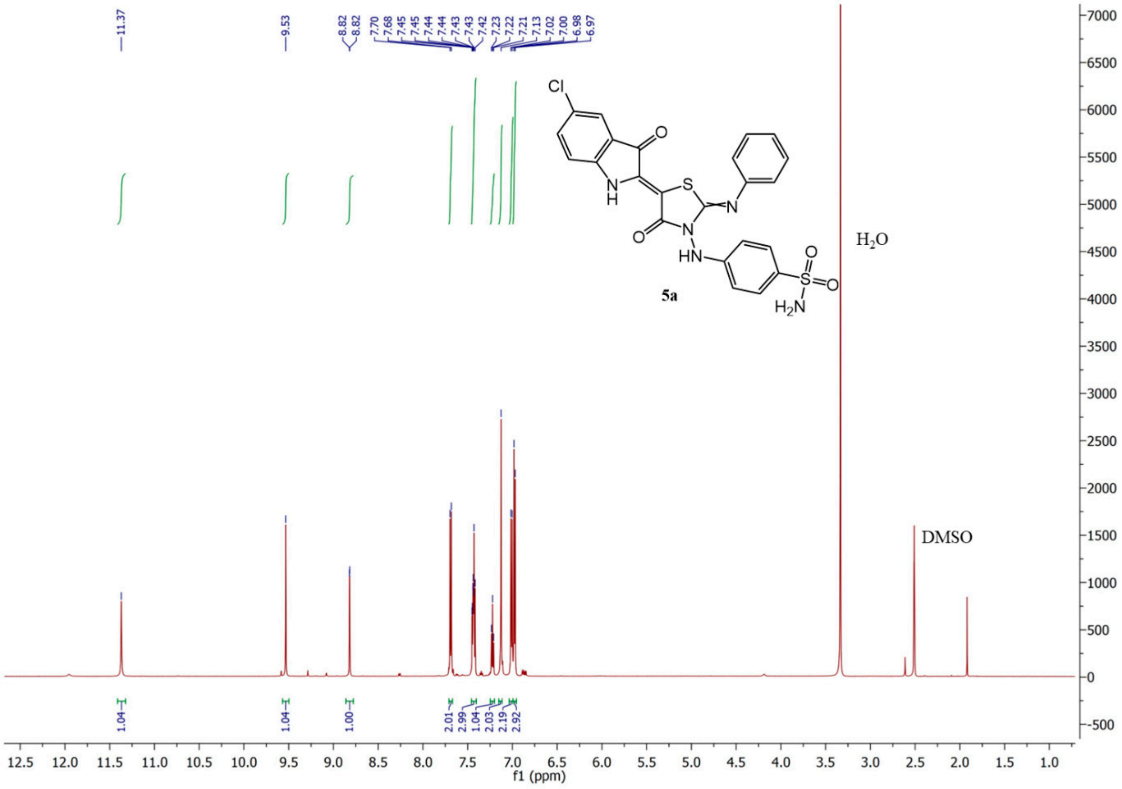

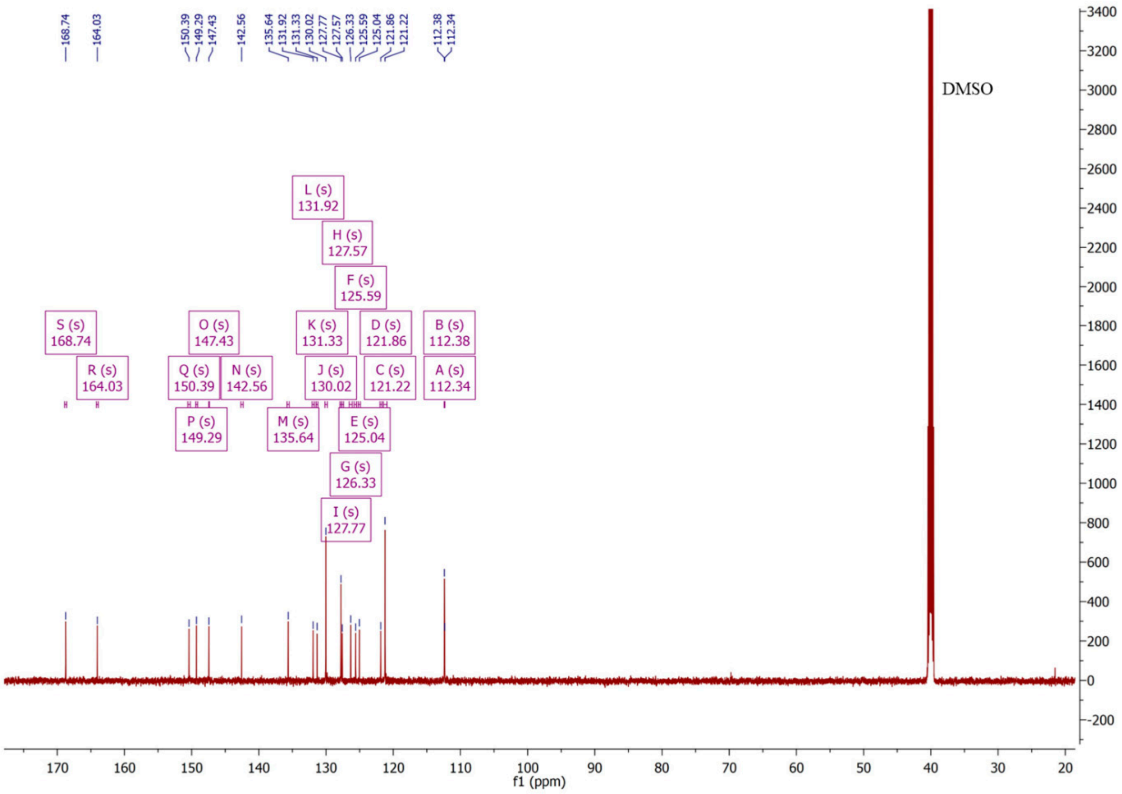

^{1}H NMR and ^{13}C NMR of compound **5b**

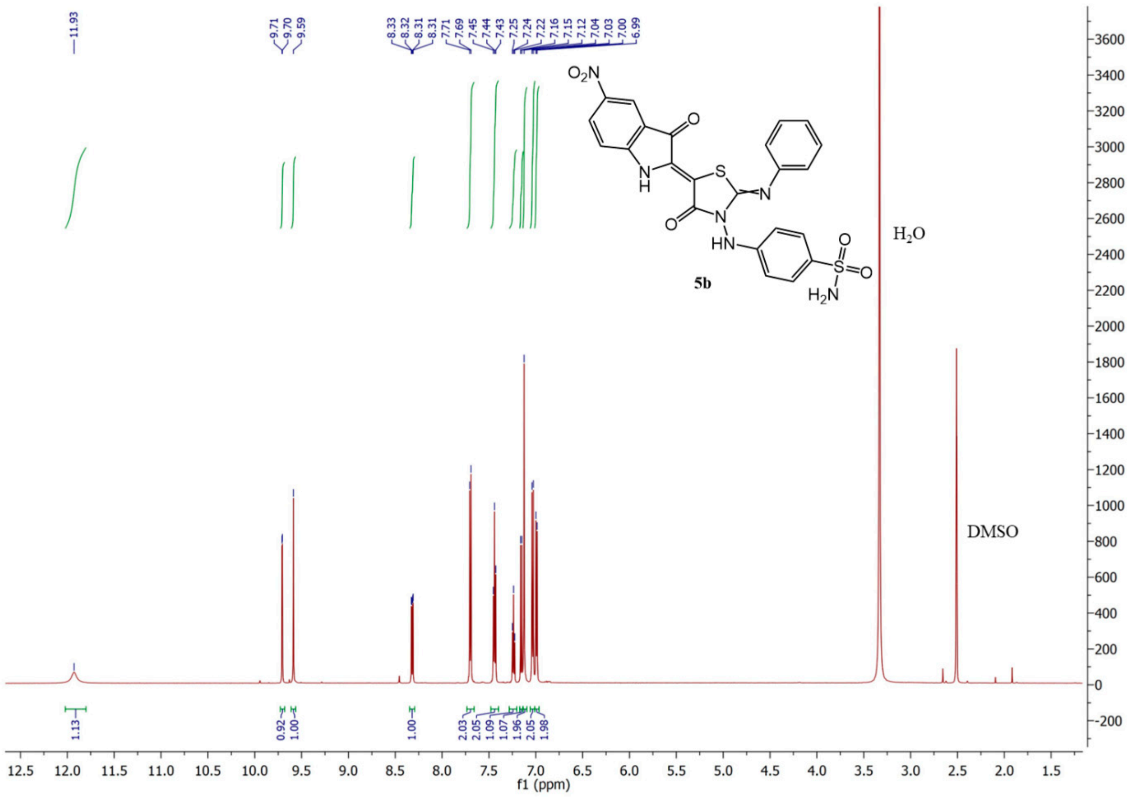

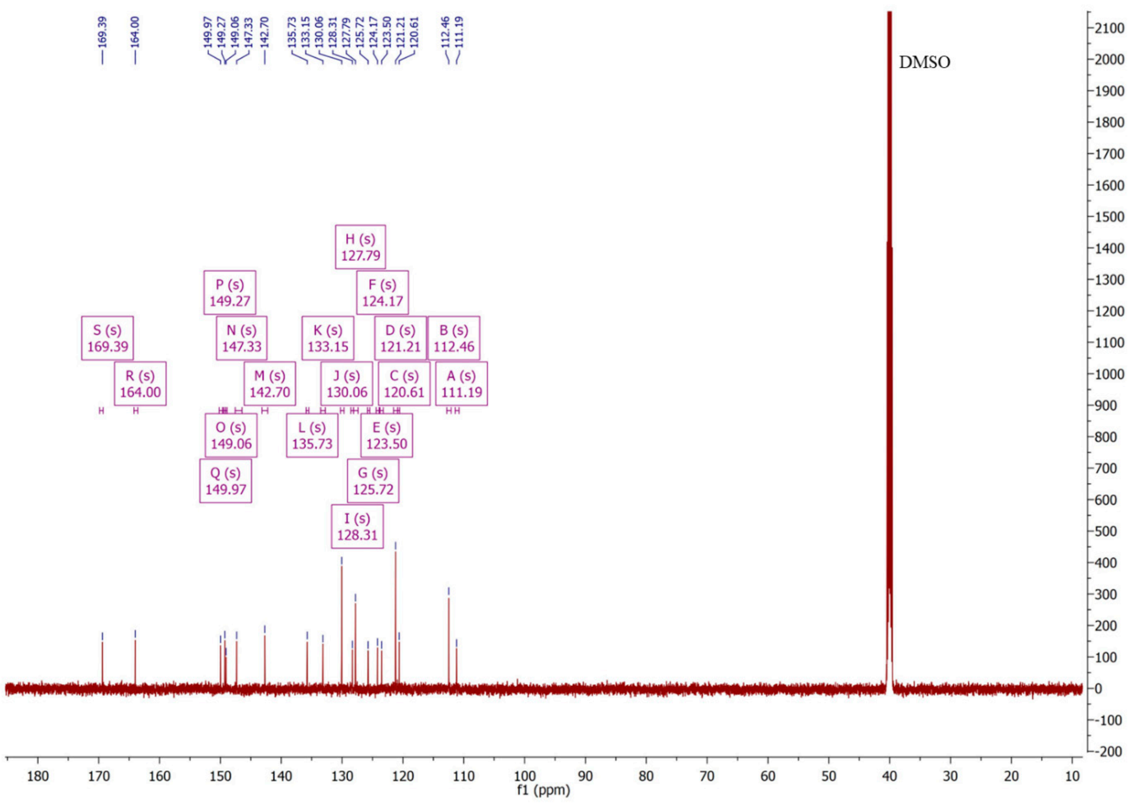

^{1}H NMR and ^{13}C NMR of compound **5c**

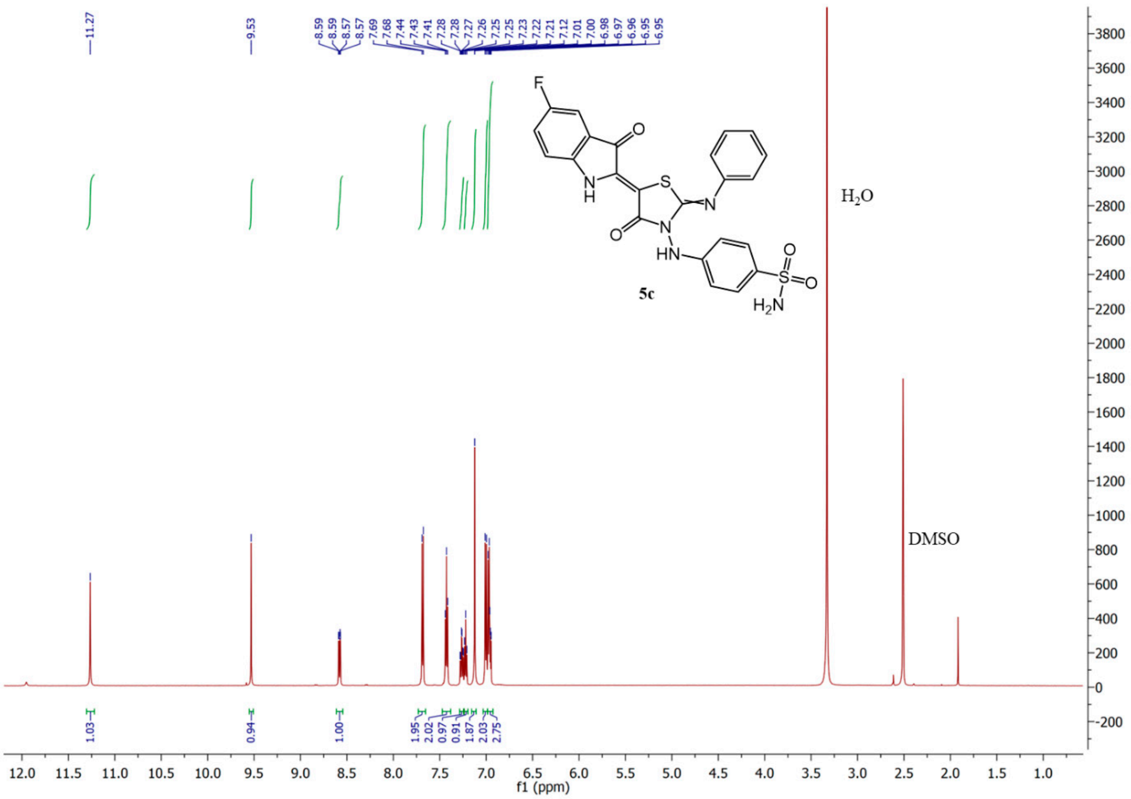

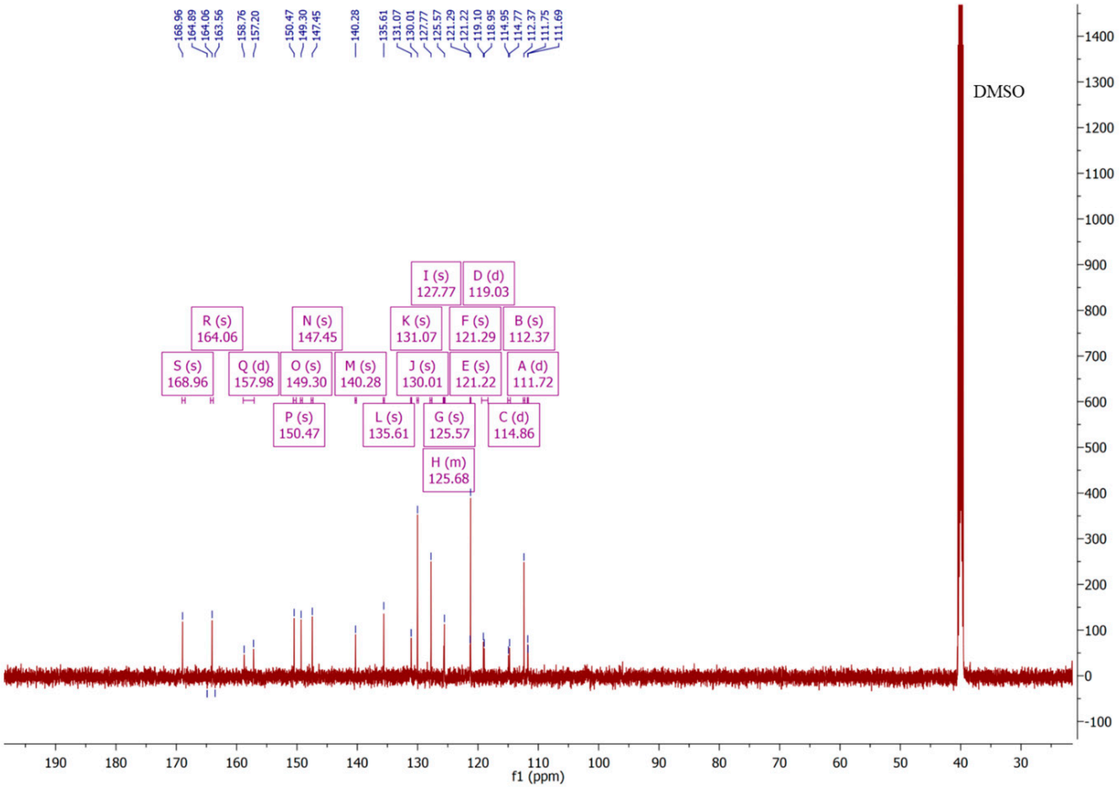

^{1}H NMR and ^{13}C NMR of compound **5d**

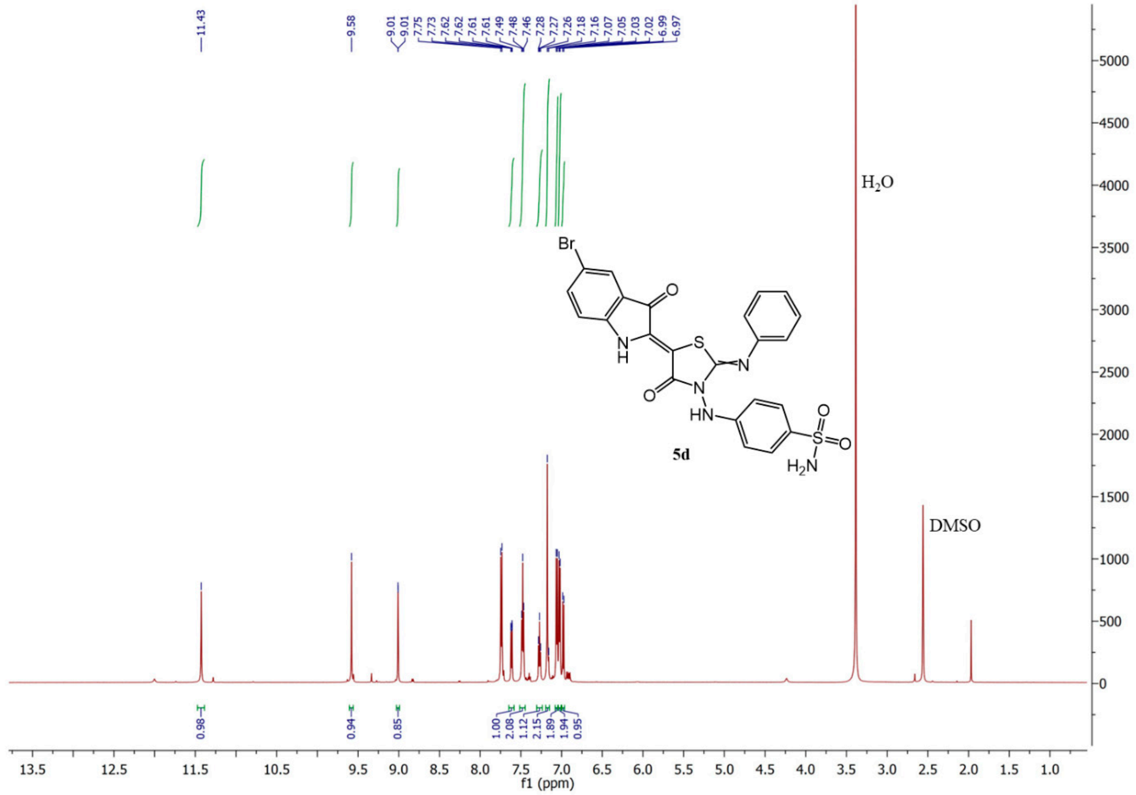

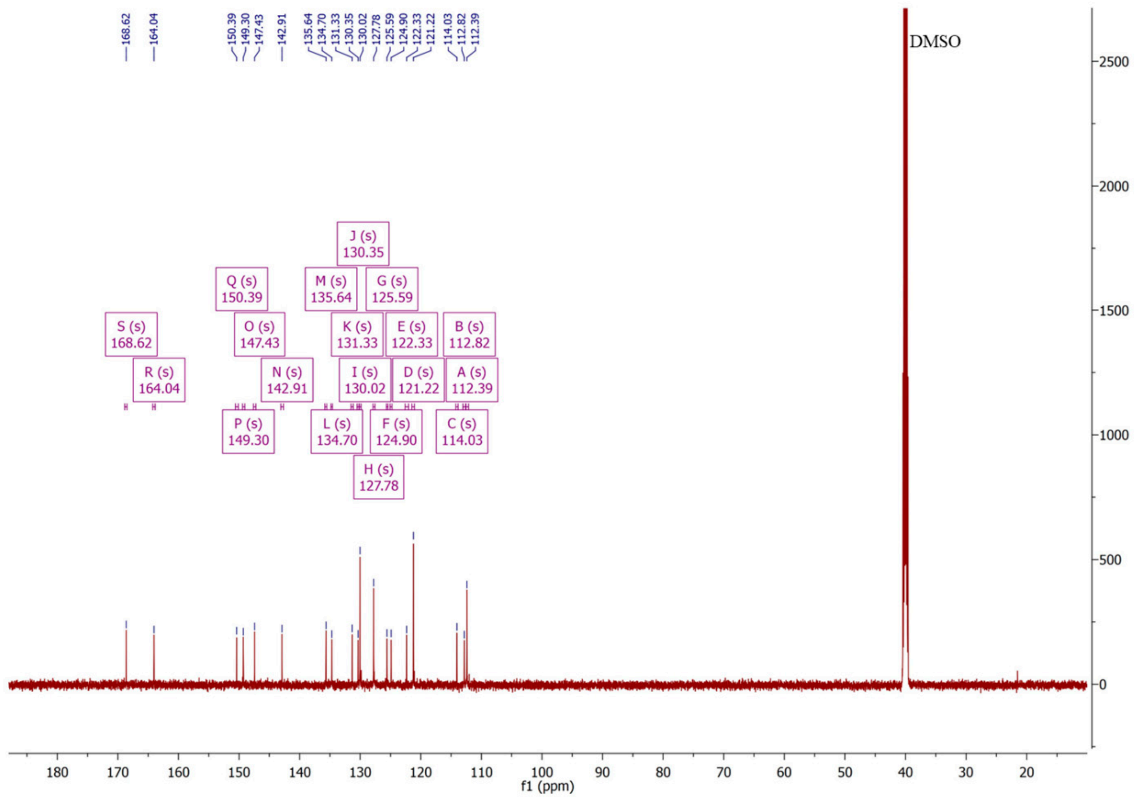

^{1}H NMR and ^{13}C NMR of compound **5e**

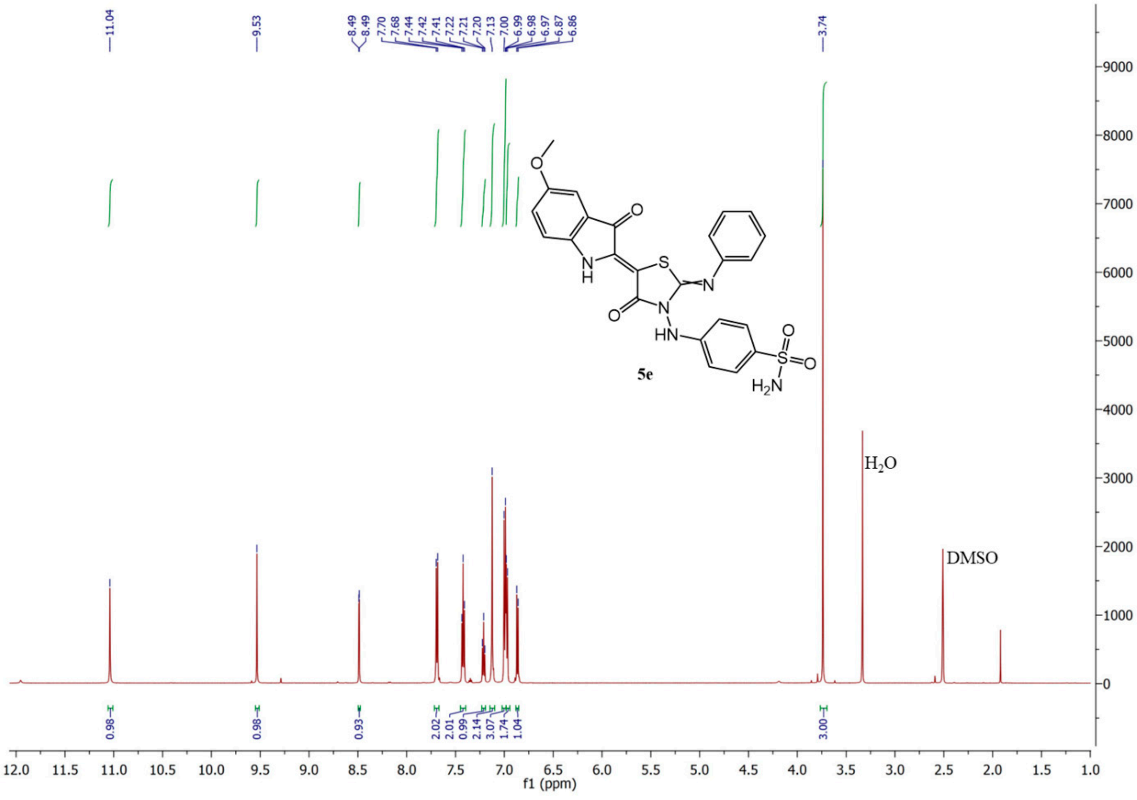

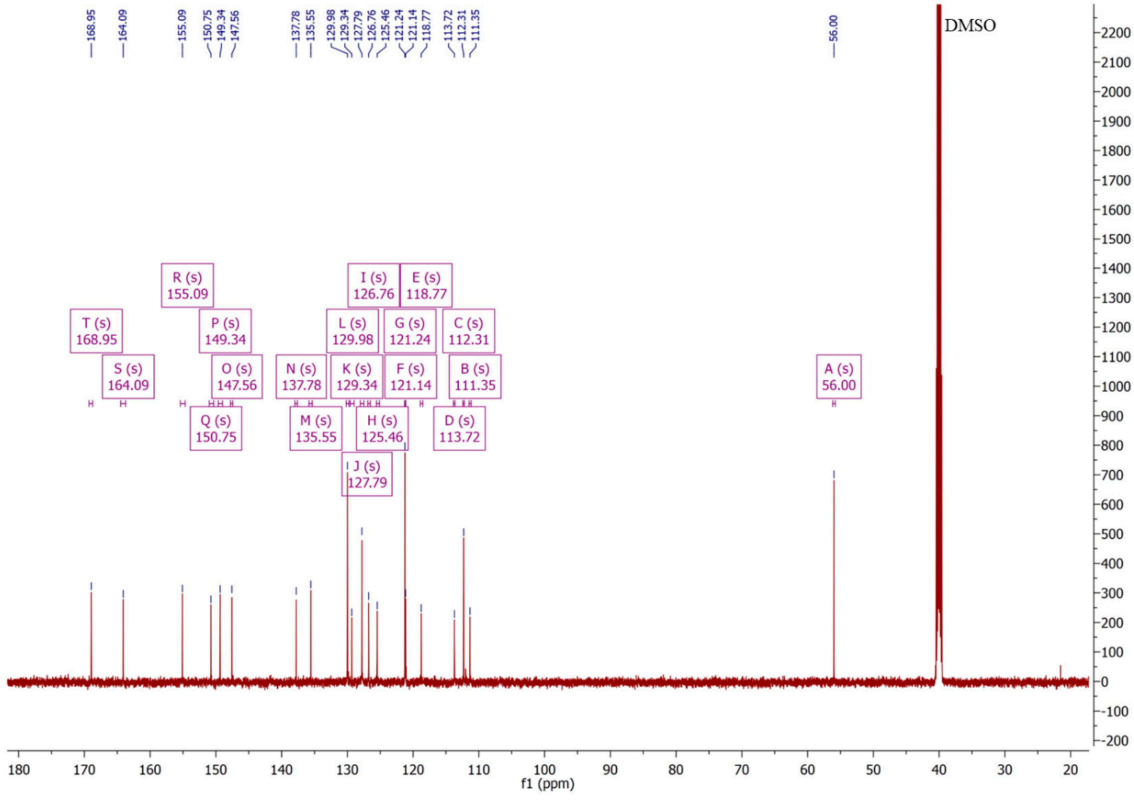

^{1}H NMR and ^{13}C NMR of compound **5f**

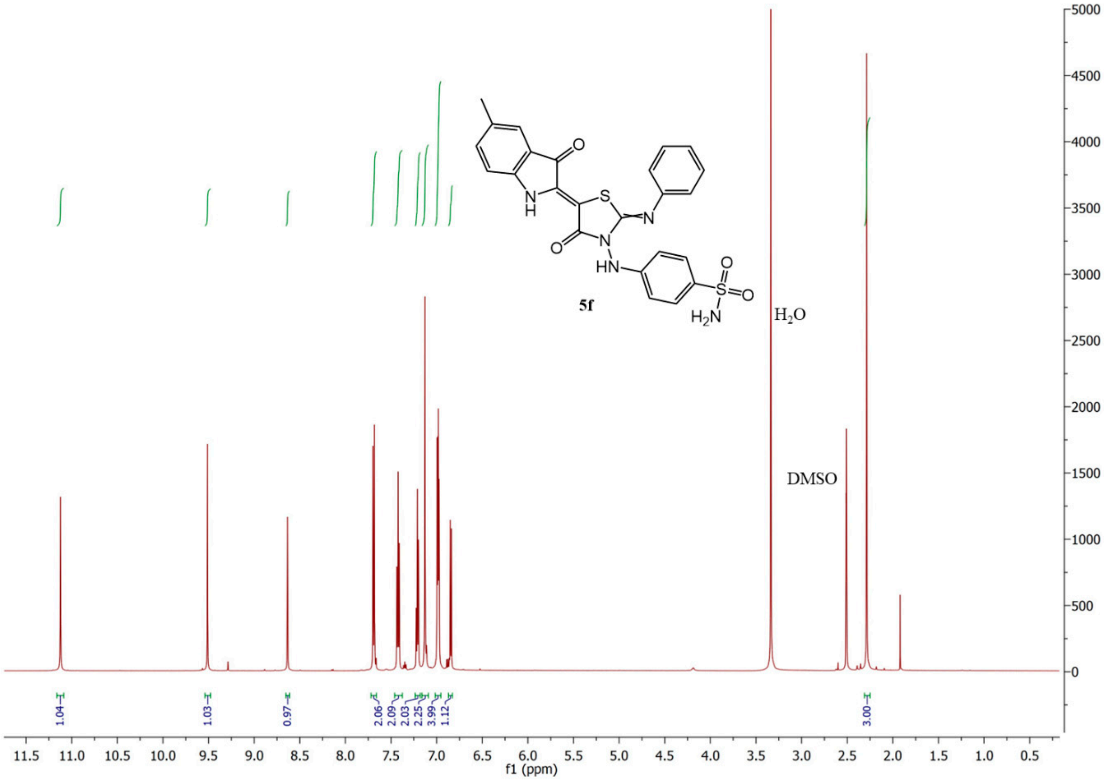

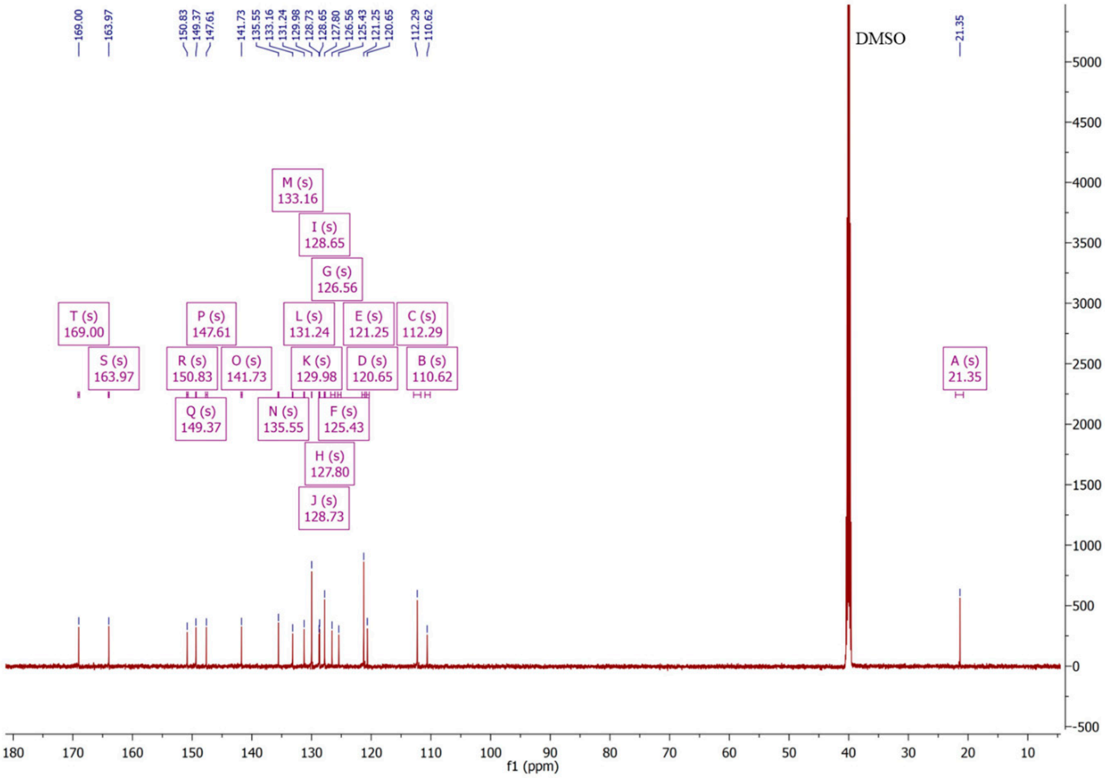

^{1}H NMR and ^{13}C NMR of compound **5g**

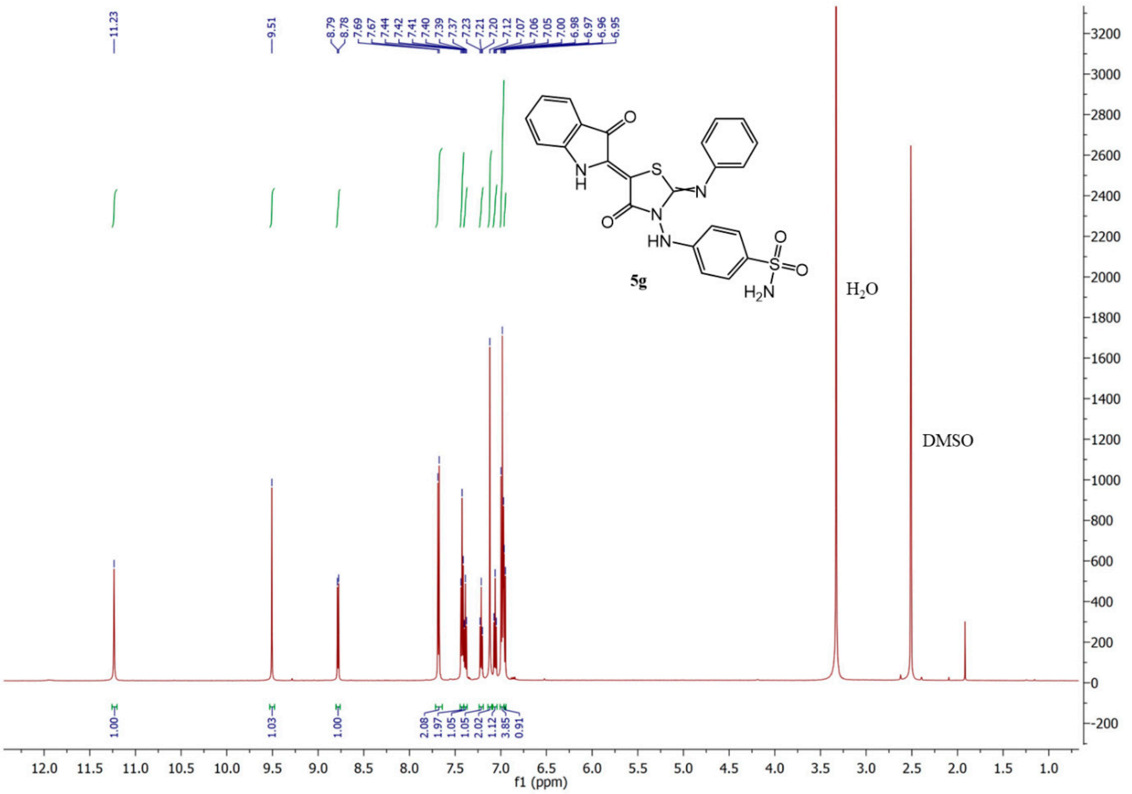

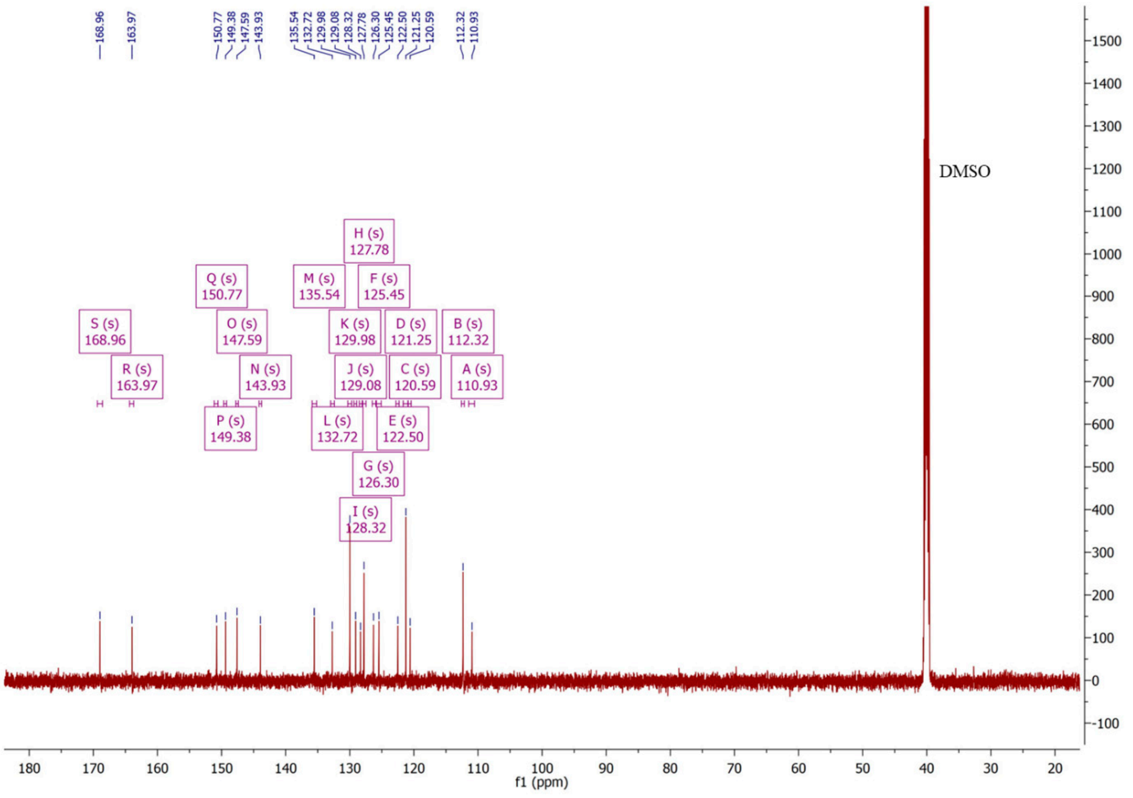

^{1}H NMR and ^{13}C NMR of compound **5h**

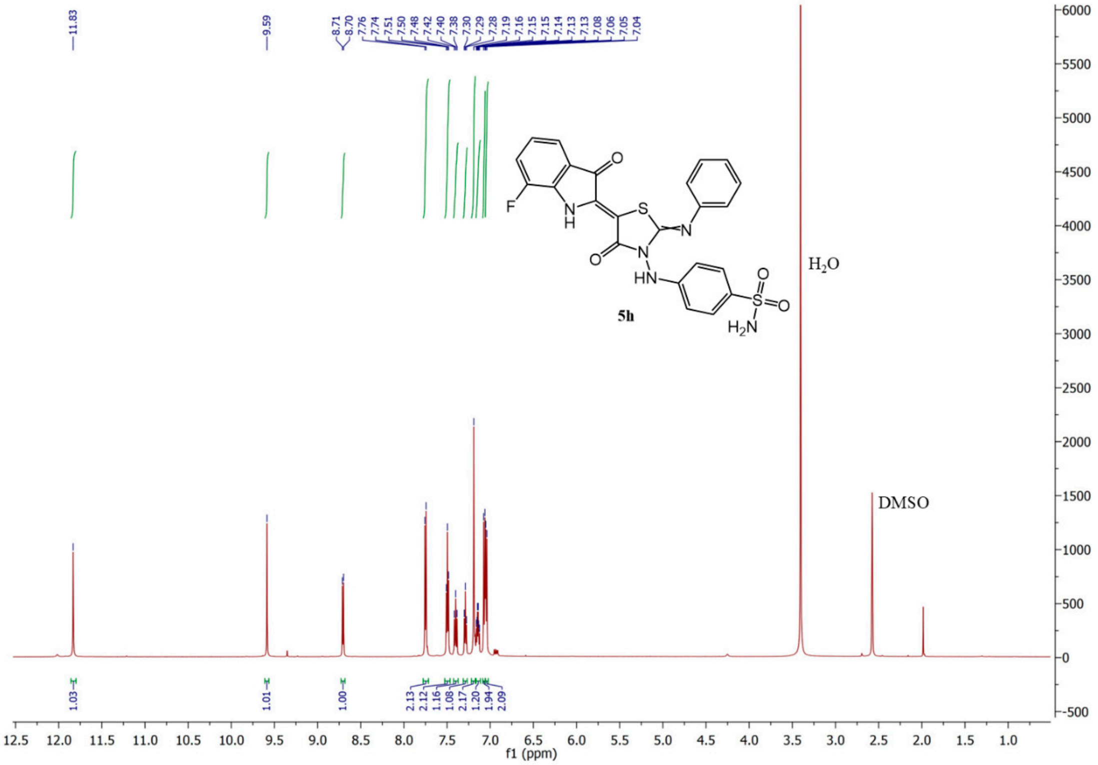

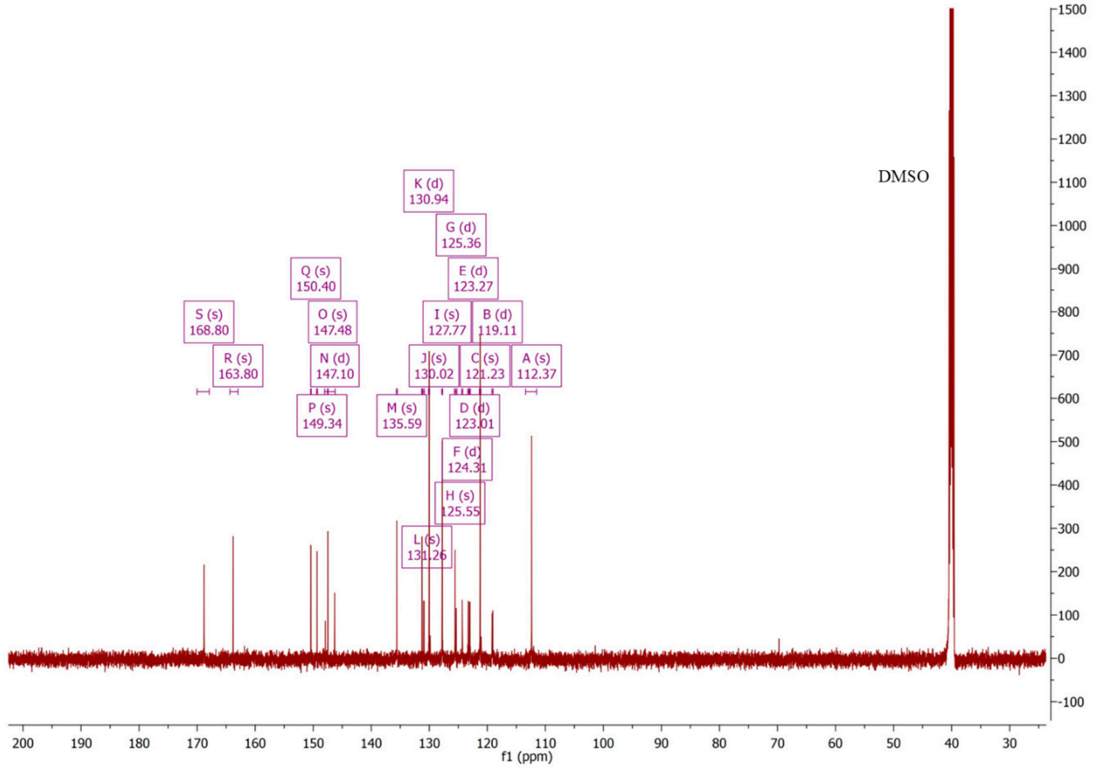

^1H NMR and $^{13}CNMR$ of compound **5i**

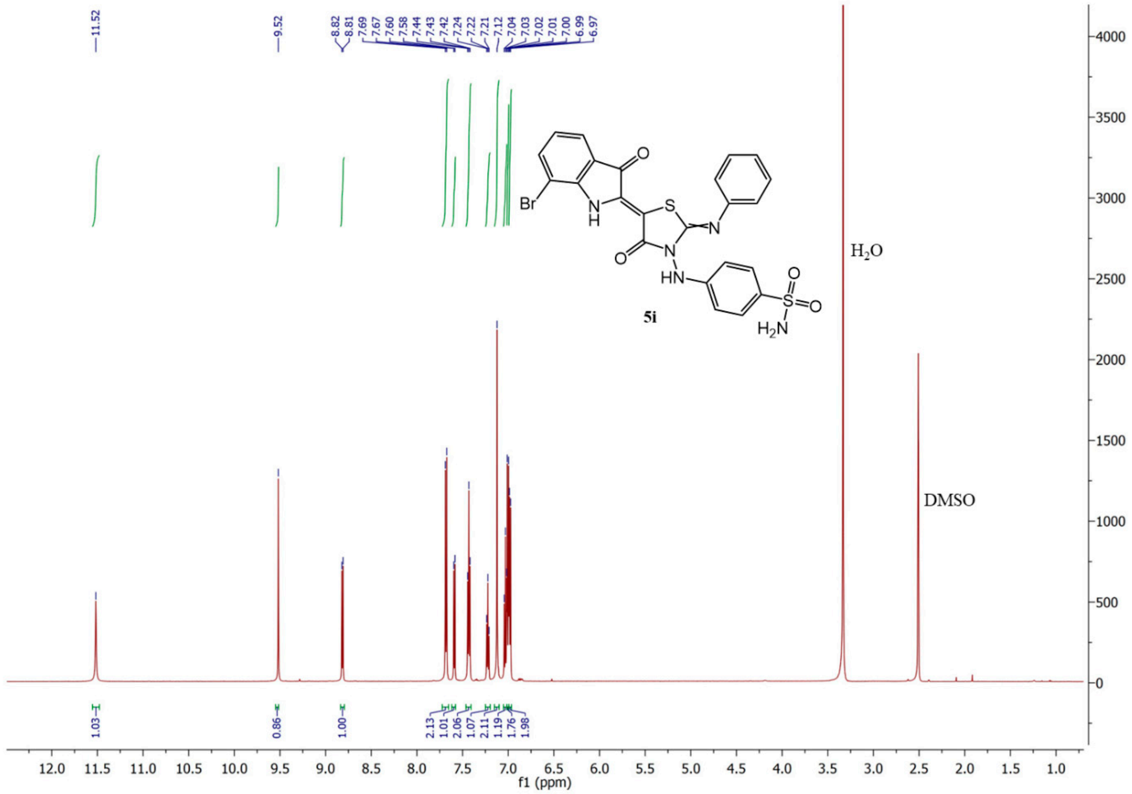

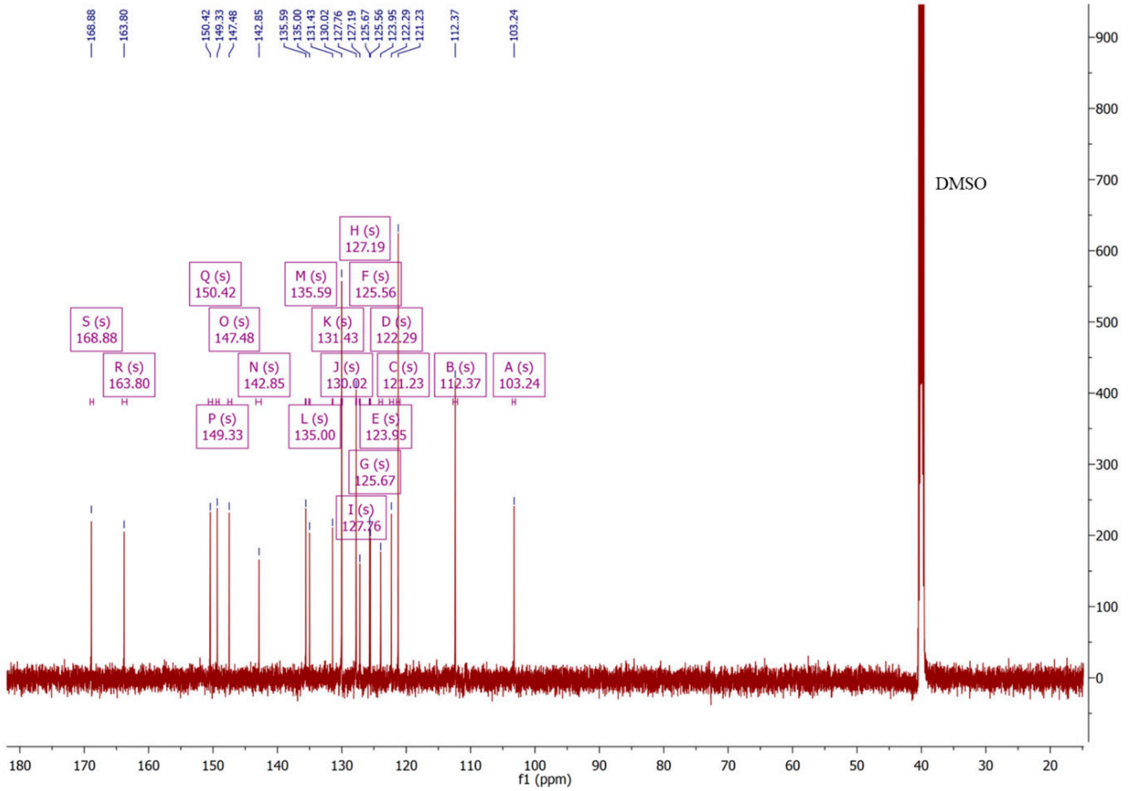

ESI-HRMS spectra of **5a-i** compounds ([M-H]$^-$)

MS spectrum of compound **5a**

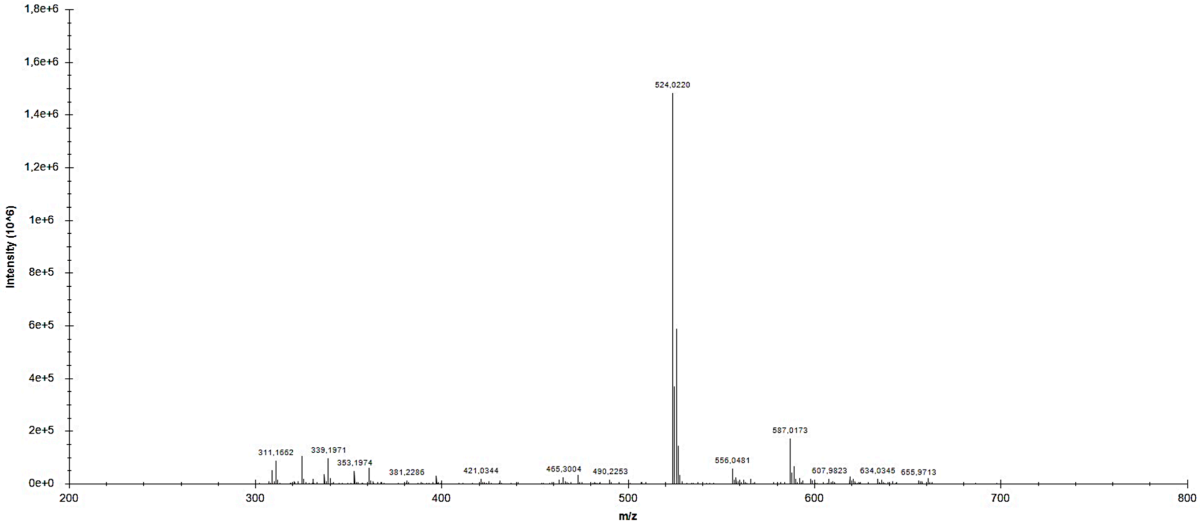

MS spectrum of compound **5b**

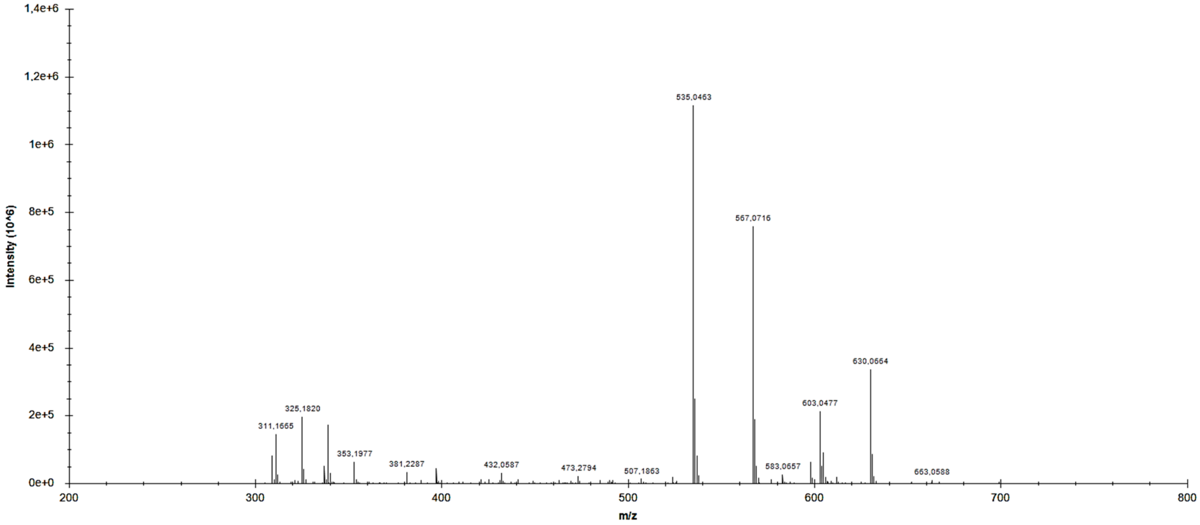

MS spectrum of compound **5c**

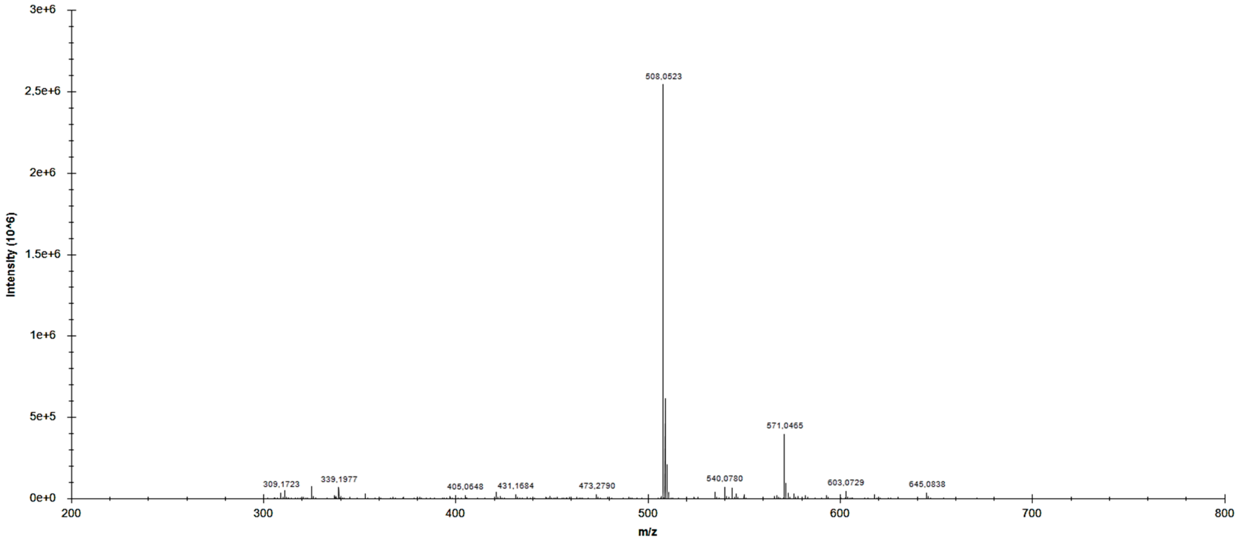

MS spectrum of compound **5d**

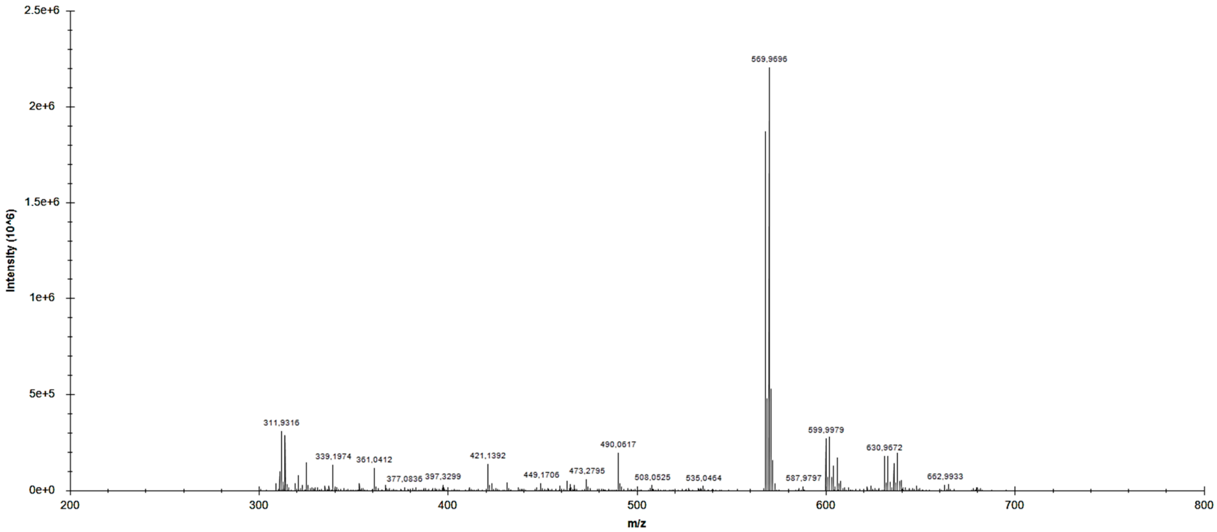

MS spectrum of compound **5e**

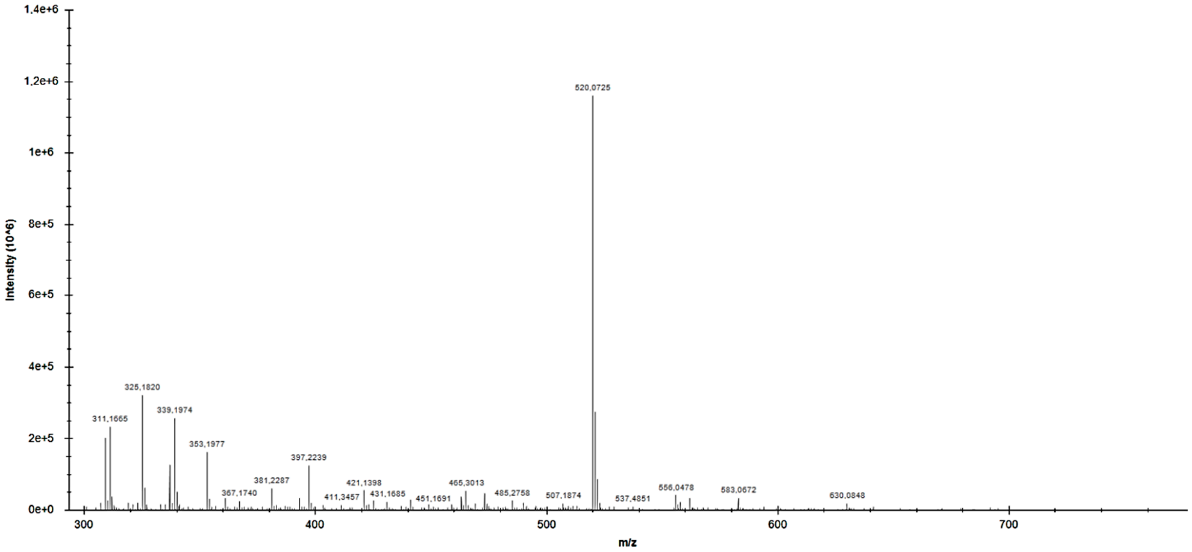

MS spectrum of compound **5f**

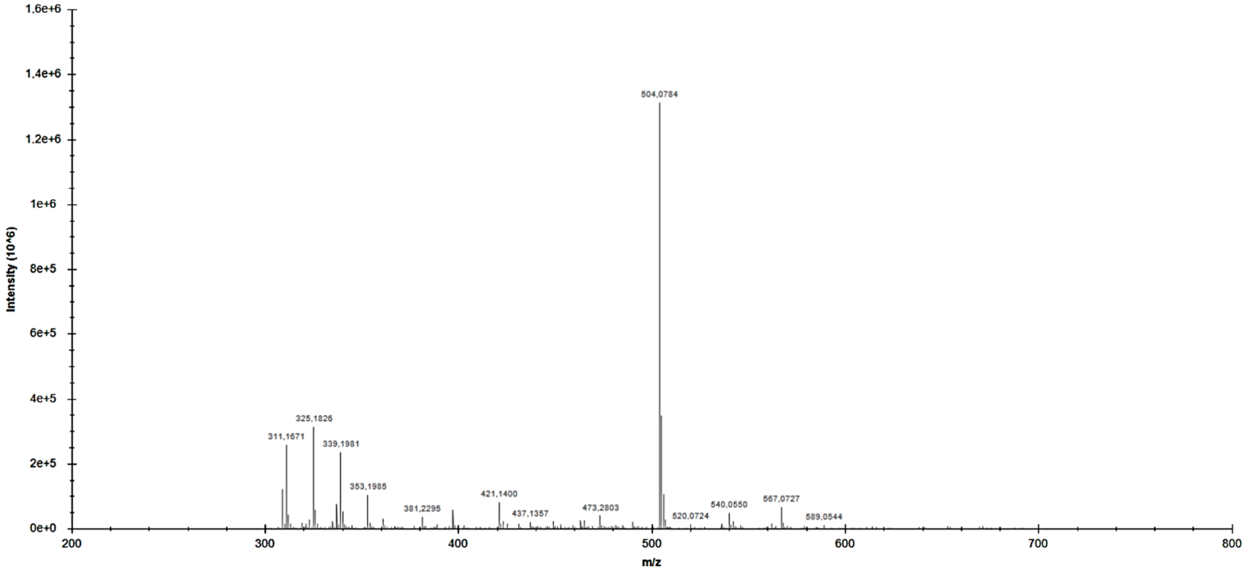

MS spectrum of compound **5g**

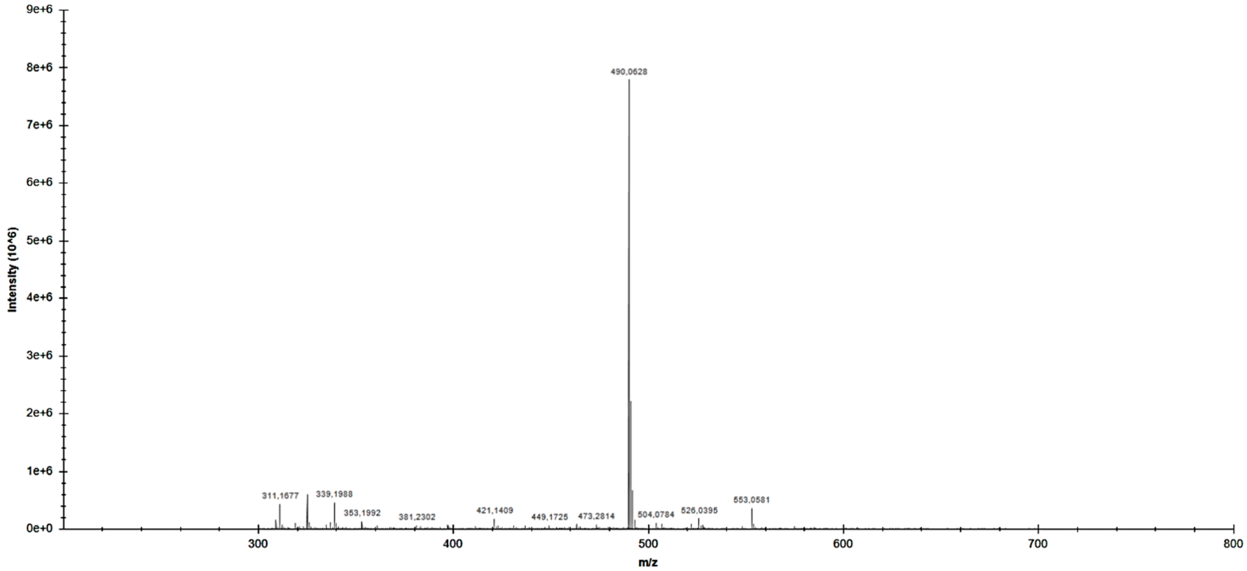

MS spectrum of compound **5h**

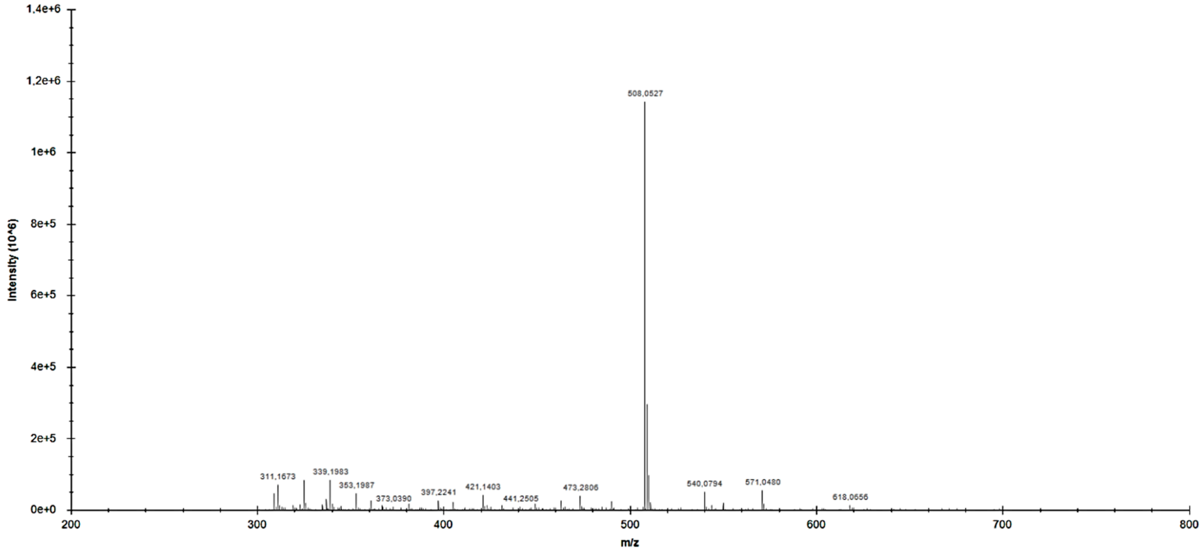

MS spectrum of compound **5i**

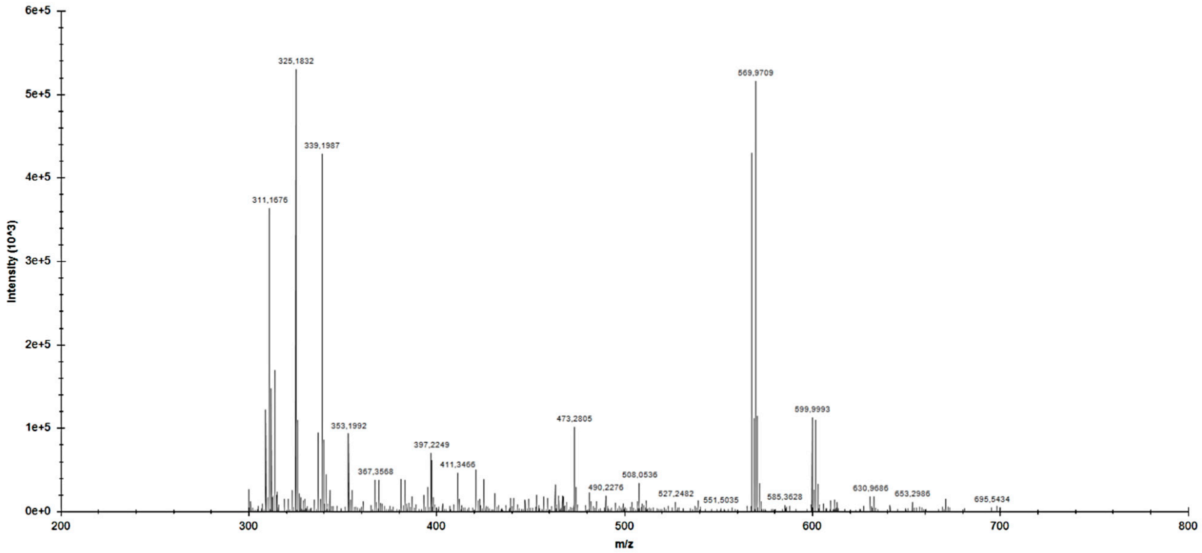

Supplement: Supplementary file 1 [file molecules-29-04444-s001.zip › molecules-3161548-supplementary.pdf]
